# Serum electrolyte concentrations and risk of atrial fibrillation: an observational and mendelian randomization study

**DOI:** 10.1186/s12864-024-10197-2

**Published:** 2024-03-16

**Authors:** Yang Wu, Xiang-Jun Kong, Ying-Ying Ji, Jun Fan, Cheng-Cheng Ji, Xu-Miao Chen, Yue-Dong Ma, An-Li Tang, Yun-Jiu Cheng, Su-Hua Wu

**Affiliations:** 1 Department of Cardiology, the First Affiliated Hospital, Sun Yat-Sen University, Guangzhou, China; 2 NHC Key Laboratory of Assisted Circulation, Sun Yat-Sen University, Guangzhou, China; 3 Department of Cardiology, Guangzhou First People’s Hospital, School of Medicine, South China University of Technology, Guangzhou, China; 4 Department of Cardiology, Guangdong Provincial People’s Hospital, Guangdong Cardiovascular Institute, Guangdong Academy of Medical Sciences, Guangzhou, China

**Keywords:** Atrial fibrillation, Serum electrolytes, Mendelian randomization analysis, Cox regression

## Abstract

**Background:**

Atrial fibrillation (AF) is a prevalent arrhythmic condition resulting in increased stroke risk and is associated with high mortality. Electrolyte imbalance can increase the risk of AF, where the relationship between AF and serum electrolytes remains unclear.

**Methods:**

A total of 15,792 individuals were included in the observational study, with incident AF ascertainment in the Atherosclerosis Risk in Communities (ARIC) study. The Cox regression models were applied to calculate the hazard ratio (HR) and 95% confidence interval (CI) for AF based on different serum electrolyte levels. Mendelian randomization (MR) analyses were performed to examine the causal association.

**Results:**

In observational study, after a median 19.7 years of follow-up, a total of 2551 developed AF. After full adjustment, participants with serum potassium below the 5th percentile had a higher risk of AF relative to participants in the middle quintile. Serum magnesium was also inversely associated with the risk of AF. An increased incidence of AF was identified in individuals with higher serum phosphate percentiles. Serum calcium levels were not related to AF risk. Moreover, MR analysis indicated that genetically predicted serum electrolyte levels were not causally associated with AF risk. The odds ratio for AF were 0.999 for potassium, 1.044 for magnesium, 0.728 for phosphate, and 0.979 for calcium, respectively.

**Conclusions:**

Serum electrolyte disorders such as hypokalemia, hypomagnesemia and hyperphosphatemia were associated with an increased risk of AF and may also serve to be prognostic factors. However, the present study did not support serum electrolytes as causal mediators for AF development.

**Supplementary Information:**

The online version contains supplementary material available at 10.1186/s12864-024-10197-2.

## Introduction

As the most common arrhythmia, the incidence and prevalence of atrial fibrillation (AF) are on the rise worldwide [1, 2]. Patients with AF are at a higher risk of stroke, heart failure (HF) and death, resulting in higher healthcare costs [3, 4]. Therefore, of supreme importance is early intervention and prevention of AF, which might reduce the societal and personal costs associated with AF.

Electrolyte disorders, including hypokalemia, hypomagnesemia, hyperphosphatemia, and hypercalcemia, are closely related to the cardiac electrophysiology due to their arrhythmogenic effect [5, 6]. Evidence is mounting to elucidate the relationship between electrolyte disturbances and AF. Extreme serum electrolyte concentrations, notably lower serum potassium and low serum magnesium levels, are associated with a higher AF risk [7, 8]. Similarly, elevated serum phosphorus concentration was also reported to be associated with a higher AF incidence, while no relationship was established between serum calcium levels and AF risk [9]. Nonetheless, no evidence exists from randomized controlled trials assessing the causal effect of serum electrolytes on AF. Additionally, ambiguity exists regarding the nature and magnitude of the prospective association between serum electrolyte concentrations and AF risk, considering that most of the previous studies were cross-sectional studies in populations with pre-existing AF. Due to potential biases such as confounders or reverse causation, the association between serum electrolytes and AF has been suggested but needs to be systematically and effectively evaluated. Moreover, whether serum electrolytes play a causal role in the development of AF also remains unclear. Given that electrolyte disorders are controllable risk factors, it will be of clinical value if serum electrolytes are shown to lead to the development of AF causally.

Mendelian randomization (MR) is a credible approach that infers the causality of risk factors (exposure) on disease (outcome) using genetic variants as instrumental variables [10]. Specifically, the random distribution of alleles from parent to offspring at conception enables genetic information independent of disease condition (reverse causality) without being susceptible to environmental confounders. This study used the two-sample MR to assess a causal relationship between serum electrolytes and AF risk without reverse causality and residual confounding factors by integrating observational and genetic epidemiology evidence.

## Methods

**Fig. 1:**
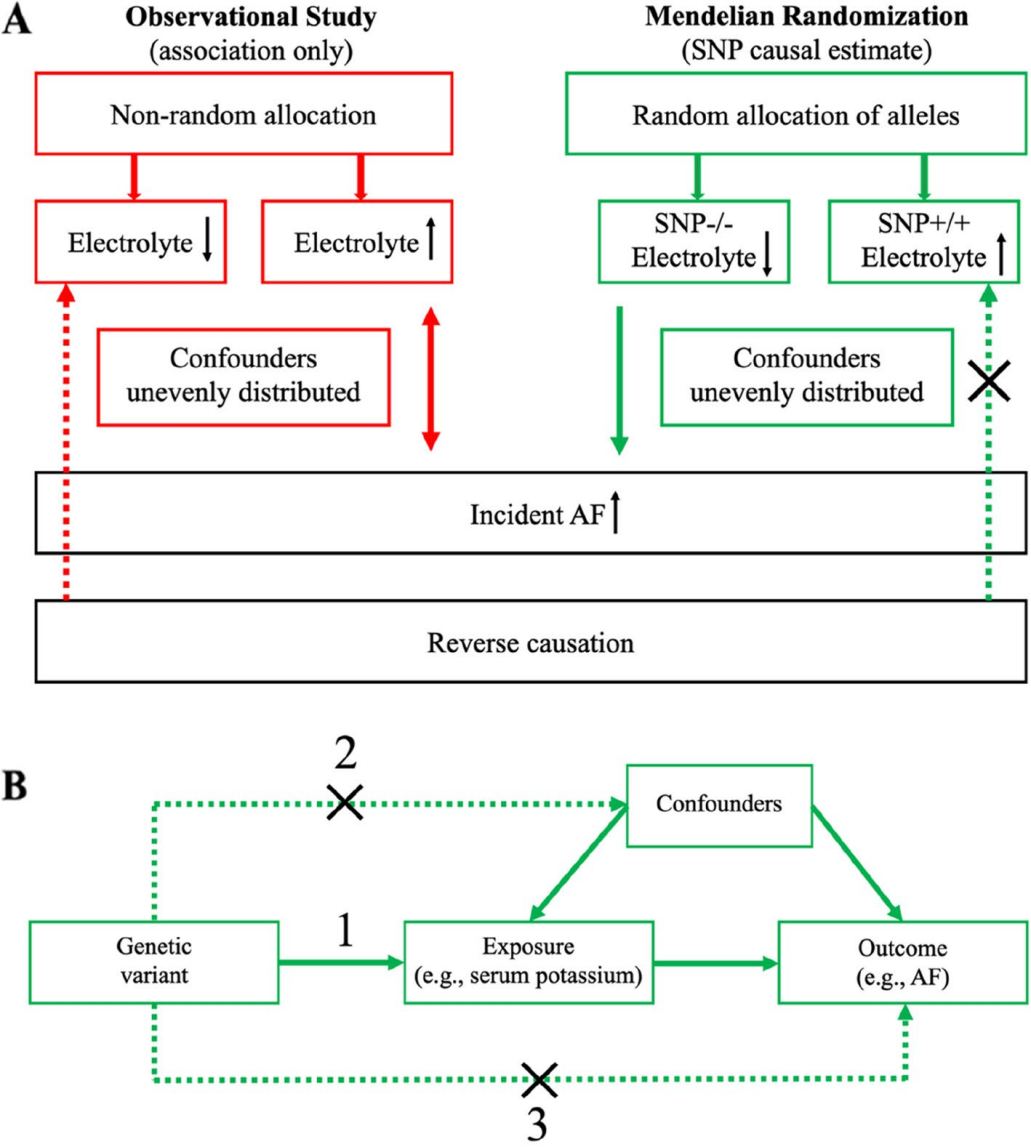
A schematic overview of the study design. **A** Comparison of observational studies and Mendelian randomization studies to help understand causality from serum electrolyte levels to high risk of AF. AF, atrial fibrillation; SNP, single-nucleotide polymorphism. **B** Mendelian randomization use genetic variant associated with exposure to estimate the causal effect of the exposure on the outcome. The three core assumptions are: [1] the genetic variant must be strongly associated with the exposure; [2] the genetic variant should be independent of any measured and unmeasured confounders; and [3] the genetic variant must influence the outcome through the exposure only and not through any direct or alternative pathways

A schematic overview of the study design is shown in Fig. 1.

### Study population

The Atherosclerosis Risk in Communities (ARIC) study is a population-based, prospective cohort study of cardiovascular risk factors in four US communities (Forsyth County, NC; Jackson, MS; suburbs of Minneapolis, MN; and Washington County, MD) [11]. The participants in the baseline period (1987 to 1989) included 15,792 of both genders aged 45 to 64 years. Study participants underwent follow-up visits in 1990–92, 1993–95, 1996–98, 2011–13, 2016–17, and 2018–19. Additionally, ARIC participants received annual follow-up calls since baseline, and survivors had a response rate of ≥ 90%. An extensive questionnaire was collected during each follow-up visit, followed by clinical examination and blood sample testing. Institutional review boards approved the ARIC study, and all participants provided informed consent.

The present analysis included a total of 15,792 participants at baseline. Participants with prevalent AF or missing follow-up data for AF (*n* = 598) were excluded. Furthermore, those with incomplete serum electrolyte data (*n* = 342) were also excluded, resulting in a final sample size of 14,852.

### Serum electrolytes measurement and covariates assessment

At the ARIC central laboratory, serum potassium was measured using an ion-selective electrode (Roche C501 Chemistry Analyzer), while serum magnesium was measured using colorimetric methods on the Roche Cobas 6000 Chemistry Analyzer (Roche Diagnostics; Indianapolis, Indiana). Serum calcium and phosphate were measured in frozen serum samples, using strategies based on o-cresolphthalein complexone and ammonium molybdate respectively. Calcium measurements were adjusted for albumin levels using the following equation: total corrected calcium = measured total calcium (mg/dL) + 0.8 [4.0 – serum albumin (g/dL)].

The C-reactive protein was measured at visit 2, and the other covariates were assessed at visit 1. The participants reported the information on age, gender, race, smoking, alcohol intake, education level, history of cardiovascular disease, and use of medications. Body mass index (BMI) was calculated as weight in kilograms divided by height in meters squared. Blood pressure was measured using a random-zero sphygmomanometer after five minutes of rest in the sitting position, and was defined as the average of the second and third measurements taken. The HF and coronary heart disease (CHD) definitions have been previously published [12, 13]. Diabetes mellitus was defined as fasting glucose ≥ 126 mg/dL, non-fasting glucose ≥ 200 mg/dL, treatment for diabetes mellitus, or self-reported physician diagnosis of diabetes. As executed in previously published ARIC studies, each exercise was converted into metabolic equivalent as per the Compendium of Physical Activities [14, 15]. High-density lipoprotein cholesterol (HDL-c) was measured using enzymatic measures, and low-density lipoprotein cholesterol (LDL-c) was calculated based on Friedewald formula [16]. The serum creatinine was measured using a modified kinetic Jaffe method. The left ventricular hypertrophy was defined as left ventricular mass index ≥ 51 g/m^2.7^ and the QT interval from the digital 12-lead electrocardiogram (ECG) was determined by the NOVACODE program [17, 18].

### AF ascertainment

Ascertainment of AF has been described previously and conducted using three methods, i.e., ECG, hospital discharge codes, and death certificates [19, 20]. At each ARIC study visit, a 12-lead ECG was performed using a MAC PC cardiograph (Marquette Electronics Inc, Milwaukee, WI) and transmitted to the ARIC ECG Reading Center for coding, interpretation and storage. The ECG recordings were computer coded and checked by a trained cardiologist at a single reading center to confirm AF diagnosis. Incident AF was identified from hospitalizations or death certificates using ICD-9-CM (International Classification of Diseases, Ninth Revision, Clinical Modification) codes 427.31 (AF) or 427.32 (atrial flutter). An AF discharge code during a hospitalization with open cardiac surgery was excluded in the ARIC Study [19].

### Mendelian randomization (MR)

Causal inference using MR relies on the instrumental variable assumptions, which require that the genetic variant must be strongly associated with the exposure, should be independent of any measured and unmeasured confounders, and must influence the outcome through the exposure only and not through any direct or alternative pathways (Fig. 1B). Genetic analysis was done using publicly available, summary-level genome-wide association study (GWAS) data. The study by Nielsen et al. was the largest GWAS for AF (OpenGWAS ID: ebi-a-GCST006414) from six contributing studies of European ancestry to date, including 60,620 cases of AF and 970,216 control subjects [21]. AF was defined based on ICD-9 code 427.3 and ICD-10 code I48. Four separate two-sample MR analysis were performed to test the potential causal associations between serum potassium (OpenGWAS ID: ukb-b-17,881), magnesium (OpenGWAS ID: ukb-b-7372), calcium (OpenGWAS ID: ukb-b-8951), and phosphate (OpenGWAS ID: ukb-d-30810_raw) with the AF risk, estimating the association results in two non-overlapping populations. GWAS data for electrolytes were derived from UK Biobank, a large, prospective cohort study that enrolled > 500,000 people across the United Kingdom from 2006 to 2010 and has received long-term follow-up [22].

We used single-nucleotide polymorphisms (SNPs) associated with four serum electrolytes from UK Biobank GWAS at genome-wide significance (*P* < 5 × 10^−6^ for potassium, magnesium and calcium; *P* < 5 × 10^−8^ for phosphate) as instruments and clumped at linkage disequilibrium R^2^ < 0.001 (Supplemental Tables 1 to 4). Complete information for data sources is detailed in Supplemental Table 5.

### Statistical analysis

Since some variables had incomplete baseline data, multiple imputation was used to impute missing data by chained Eq. [23], since it reduces the selection bias possibility and is preferable to discard observations with missing values [24]. Continuous variables are presented as means ± SD and categorical variables as percentages. The baseline characteristics of the two groups were compared using unpaired t-tests for continuous variables and Chi-square tests for categorical variables. The serum electrolyte levels were categorized into seven groups with cutoffs at the 5th, 20th, 40th, 60th, 80th, and 95th percentiles of the selected four serum electrolyte levels, and used the middle category as the reference group (i.e., 40th to 60th percentile, corresponding to the middle quintile). *P* values for trend were calculated across the quintile categories using the quintile term. Cox proportional hazards regression modeled the relationship between electrolytes and incident AF events in the ARIC study. Different models were examined to investigate the effects of various confounders on the association between serum electrolytes and AF, i.e., model 1 was adjusted for age, gender, and race, and model 2 was additionally adjusted for the variables in model 1 plus hypertension, diabetes mellitus, smoke, ethanol intake, BMI, left ventricular hypertrophy at ECG, antiarrhythmic drugs, plasma creatinine, metabolic equivalent, LDL-c, C-reactive protein and angiotensin-converting enzyme inhibitors. The results were presented as hazard ratio (HR) and 95% confidence interval (CI). Kaplan–Meier estimates were constructed to show the cumulative AF incidence risk by serum electrolyte quartiles, and differences among quartiles were compared using the log-rank test. Additionally, restricted cubic splines were used to examine the presence of a dose–response association between selected four serum electrolytes and AF. Three knots were chosen for the analysis according to Akaike’s information criterion to provide a smooth and flexible description of the dose-response relationship.

For two-sample MR, the inverse variance-weighted (IVW) method was used to estimate causal effect [25]. IVW was considered the most reliable method if there was no directional pleiotropy (P for MR-Egger intercept > 0.05) [26]. Standard sensitivity analysis, i.e., MR-Egger, weighted median, and weighted mode, was carried out to detect whether there was a violation of key assumptions underlying MR [27, 28]. An estimated intercept term of the MR-Egger regression deviating from zero indicated directional pleiotropy.

Besides, the asymmetry of the funnel plot might indicate a violation of the main MR assumptions. Leave-one-out sensitivity analysis was performed to detect if a single SNP drove an association. F-statistics were calculated to quantify the strength of the genetic instrumental variables. Effect sizes are expressed as odds ratio (OR) alongside 95% confidence intervals (CI). Cochran’s Q test was applied to assess heterogeneity between genetic variants estimates. Variance for electrolyte levels was calculated using the formula of $\mathrm R^2=\left(\mathrm\beta\times\sqrt{2\times\mathrm{MAF}\left(1-\mathrm{MAF}\right)}\right)^2$  assuming no genetic interactions, as published before [29]. F-statistics for all genetic instruments used in this study were greater than 10, indicating a low likelihood of weak instrumental variable bias.

All analysis were conducted in Stata version 16.0 (StataCorp LP, College Station, TX) and R version 4.2.1 (R Foundation for Statistical Computing). MR analysis was performed using the R-based package “TwoSampleMR” [30]. The *P* value for GWAS with genome-wide significance was set as less than 5 × 10^−6^ for potassium, magnesium, calcium and less than 5 × 10^−8^ for phosphate. A two-sided *P* value < 0.05 was considered statistically significant for all other analysis.

## Results

### Baseline characteristics in the ARIC

The clinical baseline characteristics of the 14,852 ARIC participants are listed in Table 1. Over a median follow-up of 19.7 years, 2551 (17.18%) of all participants developed incident AF. The average age (SD) of non-AF and AF patients were 53.80 (5.72) and 56.50 (5.43) years respectively. Compared to participants without incident AF, those with incident AF were inclined to be smokers, and had higher systolic blood pressure and BMI, but lower education level, HDL-c, and serum magnesium. In addition, AF patients were susceptible to HF, CHD, diabetes mellitus, and left ventricular hypertrophy. There were no statistical differences in alcohol consumption, metabolic equivalent, LDL-c, potassium, phosphate, and calcium levels between the two groups.

**Table 1:** Baseline characteristics of 14,852 participants

	Incident Atrial Fibrillation		
Characteristic	No (*n* = 12,301)	Yes (*n* = 2,551)	*P* Value
Age, mean (SD), years	53.80 (5.72)	56.50 (5.43)	< 0.001
Sex, N, (%)			< 0.001
Female	6,880 (55.93)	1,195 (46.84)	
Male	5,421 (44.07)	1,356 (53.16)	
Race, N, (%)			< 0.001
Black	3,342 (27.17)	471 (18.46)	
White	8,959 (72.83)	2,080 (81.54)	
Systolic BP, mean (SD), mmHg	120.66 (18.86)	125.14 (19.66)	< 0.001
Diastolic BP, mean (SD), mmHg	73.67 (11.25)	73.88 (11.78)	0.40
History of hypertension, N (%)	6,230 (50.65)	1550 (60.76)	< 0.001
Education level, N (%)			< 0.001
Low	2,831 (23.01)	696 (27.28)	
Intermediate	5,038 (40.96)	1,044 (40.93)	
High	4,432 (36.03)	811 (31.79)	
Smoking, N, (%)			< 0.001
Never smoker	5,184 (42.14)	913 (35.79)	
Past smoker	3,920 (31.87)	901 (35.32)	
Current smoker	3,197 (25.99)	737 (28.89)	
History of HF, N (%)	519 (4.22)	206 (8.08)	< 0.001
History of CHD, N (%)	521 (4.24)	224 (8.78)	< 0.001
Diabetes mellitus, N (%)	1,377 (11.19)	421 (16.50)	< 0.001
Alcohol consumption, N (%)	6,907 (56.15)	1,433 (56.17)	0.98
MET, mean (SD), hours/week	3.28 (3.02)	3.25 (2.97)	0.65
HDL-c, mean (SD), mg/dL	1.35 (0.44)	1.25 (0.42)	< 0.001
LDL-c, mean (SD), mg/dL	3.56 (1.02)	5.60 (1.00)	0.10
BMI, mean (SD), kg/m^2^	27.50 (5.26)	28.75 (5.72)	< 0.001
QTc interval, mean (SD), msec	415.81 (19.45)	419.47 (21.20)	< 0.001
LVH, N (%)	244 (1.98)	87 (3.41)	< 0.001
Antiarrhythmic drugs, N (%)	68 (0.55)	49 (1.92)	< 0.001
plasma creatinine, mean (SD), mg/dL	1.11 (0.44)	1.13 (0.42)	0.02
Serum potassium, mean (SD), mmol/L	4.43 (0.48)	4.42 (0.49)	0.75
Serum magnesium, mean (SD), mg/dL	1.63 (0.16)	1.62 (0.17)	< 0.001
Serum phosphate, mean (SD), mg/dL	3.43 (0.49)	3.42 (0.52)	0.20
Serum calcium, mean (SD), mg/dL	9.89 (0.42)	9.89 (0.42)	0.42

Values are expressed as mean (SD) for continuous variables, and n (%) for categorical variables. Serum calcium was corrected for serum albumin. *BP* blood pressure, *HF* heart failure, *CHD* coronary heart disease, *MET* metabolic equivalent, *HDL-c* high-density lipoprotein cholesterol, *LDL-c* low-density lipoprotein cholesterol, *BMI* body mass index, *LVH* left ventricular hypertrophy

### Observational analysis

Compared to the middle quintile (40 to < 60th), subjects below the 5th percentile of the serum potassium had increased AF risk after adjustment for age, gender, and race (adjusted model 1, HR: 1.56; 95% CI: 1.29 to 1.89; *P* < 0.001, Table 2). A similar association between low serum potassium and higher AF risk was observed after further adjustment for multiple risk factors (adjusted model 2, HR < 5th compared to 40 to < 60th: 1.37; 95% CI: 1.13 to 1.66; *P* = 0.001; P for trend < 0.001, Table 2).

**Table 2:** Hazard ratio (95%CI) of atrial fibrillation with different electrolytes levels

		Range, mmol/L; mg/dL	No. of events (%)	Unadjusted		Adjusted model 1^a^		Adjusted model 2^b^	
				HR (95% CI)	*P* value	HR (95% CI)	*P* value	HR (95% CI)	*P* value
Potassium	< 5th	2.50–3.59	146 (19.84)	1.38 (1.15,1.66)	0.001	1.56 (1.29,1.89)	< 0.001	1.37 (1.13,1.66)	0.001
	5 to < 20th	3.60–4.09	386 (17.48)	1.05 (0.92,1.21)	0.45	1.12 (0.98,1.29)	0.11	1.08 (0.94,1.24)	0.25
	20 to < 40th	4.10–4.29	493 (16.75)	1.01 (0.89,1.14)	0.93	1.03 (0.91,1.17)	0.61	1.04 (0.91,1.18)	0.57
	40 to < 60th	4.30–4.49	489 (16.60)	1.00 (Reference)	–	1.00 (Reference)	–	1.00 (Reference)	–
	60 to < 80th	4.50–4.79	506 (17.19)	1.07 (0.94,1.21)	0.32	1.01 (0.89,1.14)	0.91	1.01 (0.89,1.15)	0.85
	80 to < 95th	4.80–5.19	394 (17.84)	1.09 (0.96,1.25)	0.19	1.04 (0.90,1.19)	0.61	1.06 (0.93,1.21)	0.40
	≥ 95th	5.20–6.50	125 (16.98)	1.08 (0.89,1.32)	0.44	1.00 (0.82,1.21)	0.96	1.02 (0.84,1.25)	0.83
	P for trend			0.002		< 0.001		< 0.001	
Magnesium	< 5th	0.50–1.39	166 (22.55)	1.76 (1.48,2.11)	< 0.001	1.85 (1.54,2.21)	< 0.001	1.43 (1.19,1.72)	< 0.001
	5 to < 20th	1.40–1.49	420 (19.02)	1.25 (1.09,1.43)	0.17	1.36 (1.19,1.55)	< 0.001	1.22 (1.06,1.39)	0.004
	20 to < 40th	1.50–1.59	492 (16.71)	1.01 (0.89,1.14)	0.93	1.01 (0.89,1.14)	0.90	0.98 (0.87,1.12)	0.80
	40 to < 60th	1.60–1.69	500 (16.98)	1.00 (Reference)	–	1.00 (Reference)	–	1.00 (Reference)	–
	60 to < 80th	1.70–1.79	495 (16.81)	1.00 (0.88,1.13)	0.96	0.94 (0.83,1.06)	0.31	0.97 (0.85,1.10)	0.59
	80 to < 95th	1.80–1.89	338 (15.31)	0.88 (0.77,1.02)	0.09	0.84 (0.73,0.96)	0.01	0.89 (0.78,1.03)	0.11
	≥ 95th	1.90–3.10	128 (17.39)	1.01 (0.83,1.23)	0.90	0.95 (0.78,1.16)	0.64	0.97 (0.80,1.19)	0.78
	P for trend			< 0.001		< 0.001		< 0.001	
Phosphate	< 5th	1.00-2.59	153 (20.79)	1.26 (1.05,1.52)	0.01	1.07 (0.89,1.29)	0.48	1.00 (0.83,1.21)	0.97
	5 to < 20th	2.60–2.99	397 (17.98)	1.10 (0.96,1.26)	0.16	1.01 (0.88,1.16)	0.92	0.98 (0.85,1.12)	0.73
	20 to < 40th	3.00-3.29	491 (16.68)	1.02 (0.90,1.16)	0.79	0.99 (0.87,1.13)	0.89	0.99 (0.87,1.12)	0.83
	40 to < 60th	3.30–3.49	478 (16.23)	1.00 (Reference)	–	1.00 (Reference)	–	1.00 (Reference)	–
	60 to < 80th	3.50–3.79	513 (17.43)	1.07 (0.94,1.21)	0.32	1.13 (0.99,1.28)	0.06	1.12 (0.99,1.27)	0.08
	80 to < 95th	3.80–4.19	383 (17.35)	1.09 (0.95,1.25)	0.23	1.20 (1.05,1.38)	0.01	1.18 (1.03,1.36)	0.02
	≥ 95th	4.20–9.10	124 (16.85)	1.14 (0.93,1.39)	0.20	1.33 (1.09,1.63)	0.01	1.32 (1.07,1.62)	0.01
	P for trend			< 0.001		< 0.001		< 0.001	
Calcium	< 5th	7.28–9.23	122 (16.58)	0.86 (0.71,1.06)	0.15	0.87 (0.71,1.07)	0.18	0.90 (0.74,1.11)	0.33
	5 to < 20th	9.24–9.53	364 (16.49)	0.89 (0.77,1.02)	0.08	0.90 (0.79,1.03)	0.13	0.91 (0.79,1.04)	0.16
	20 to < 40th	9.54–9.77	497 (16.88)	0.94 (0.83,1.06)	0.33	0.97 (0.86,1.10)	0.68	0.97 (0.86,1.10)	0.67
	40 to < 60th	9.78–9.97	512 (17.39)	1.00 (Reference)	–	1.00 (Reference)	–	1.00 (Reference)	–
	60 to < 80th	9.98–10.21	540 (18.34)	1.09 (0.96,1.23)	0.19	1.10 (0.97,1.24)	0.13	1.07 (0.95,1.22)	0.25
	80 to < 95th	10.22–10.57	376 (17.03)	1.05 (0.91,1.20)	0.51	1.10 (0.96,1.26)	0.18	1.03 (0.90,1.18)	0.65
	≥ 95th	10.58–13.56	128 (17.39)	1.17 (0.96,1.43)	0.12	1.20 (0.98,1.47)	0.07	1.08 (0.88,1.32)	0.45
	P for trend			0.02		0.02		0.50	

^a^Adjusted by age, gender and race

^b^Adjusted by model 1 + hypertension, diabetes mellitus, smoke, drink, body mass index, left ventricular hypertrophy at ECG, antiarrhythmic drugs, plasma creatinine, metabolic equivalent, low-density lipoprotein cholesterol, C-reactive protein and angiotensin-converting enzyme inhibitors

Serum magnesium was inversely associated with AF risk after primary adjustment (adjusted model 1, HR 5 to < 20th compared to 40 to < 60th: 1.36; 95% CI: 1.19 to 1.55; *P* < 0.001; HR < 5th compared with 40 to < 60th: 1.85; 95% CI: 1.54 to 2.21; *P* < 0.001, Table 2). A similar association between low serum magnesium and higher AF risk was observed after full adjustment (adjusted model 2, HR 5 to < 20th compared to 40 to < 60th: 1.22; 95% CI: 1.06 to 1.39; *P* = 0.004; HR < 5th compared to 40 to < 60th: 1.43; 95% CI: 1.19 to 1.72; *P* < 0.001; P for trend < 0.001, Table 2).

In the adjusted model 1, higher serum phosphate percentiles were associated with an increased risk of AF (adjusted model 1 h 80 to < 95th compared to 40 to < 60th: 1.20; 95% CI: 1.05 to 1.38; *P* = 0.01; HR ≥ 95th compared to 40 to < 60th: 1.33; 95% CI: 1.09 to 1.63; *P* = 0.01, Table 2). The association between higher serum phosphate percentiles and the risk of AF remained after full adjustment (adjusted model 2, HR 80 to < 95th compared to 40 to < 60th: 1.18; 95% CI: 1.03 to 1.36; *P* = 0.02; HR ≥ 95th compared to 40 to < 60th: 1.32; 95% CI: 1.07 to 1.62; *P* = 0.01; P for trend < 0.001, Table 2).

There was no evidence of an association between serum calcium and the AF risk (Table 2).

**Fig. 2:**
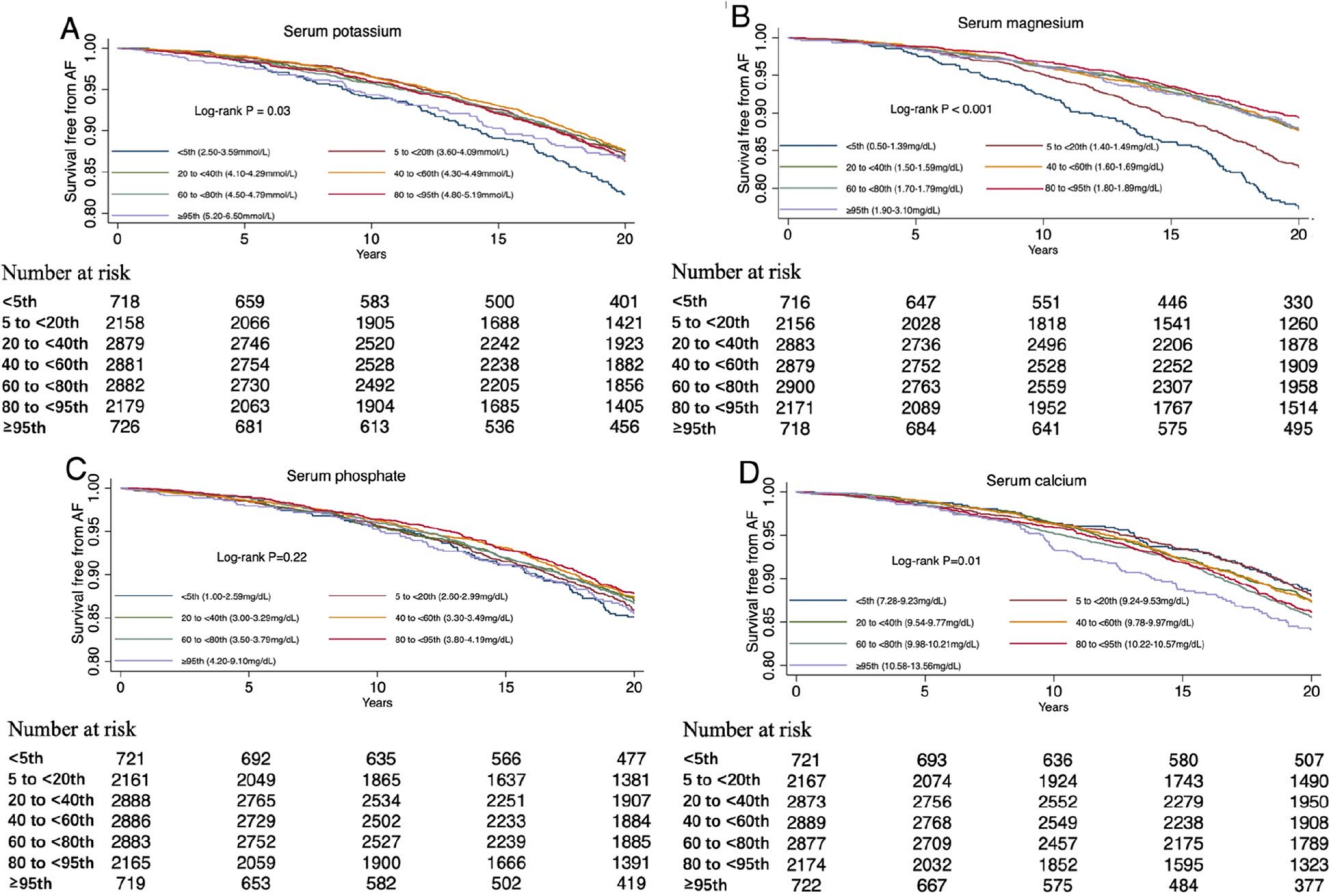
Kaplan-Meier survival curves of AF by serum electrolyte groups.** A** AF survival curves by groups of serum potassium. **B** AF survival curves by groups of serum magnesium. **C** AF survival curves by groups of serum phosphate. **D** AF survival curves by groups of serum calcium. AF, atrial fibrillation

**Fig. 3:**
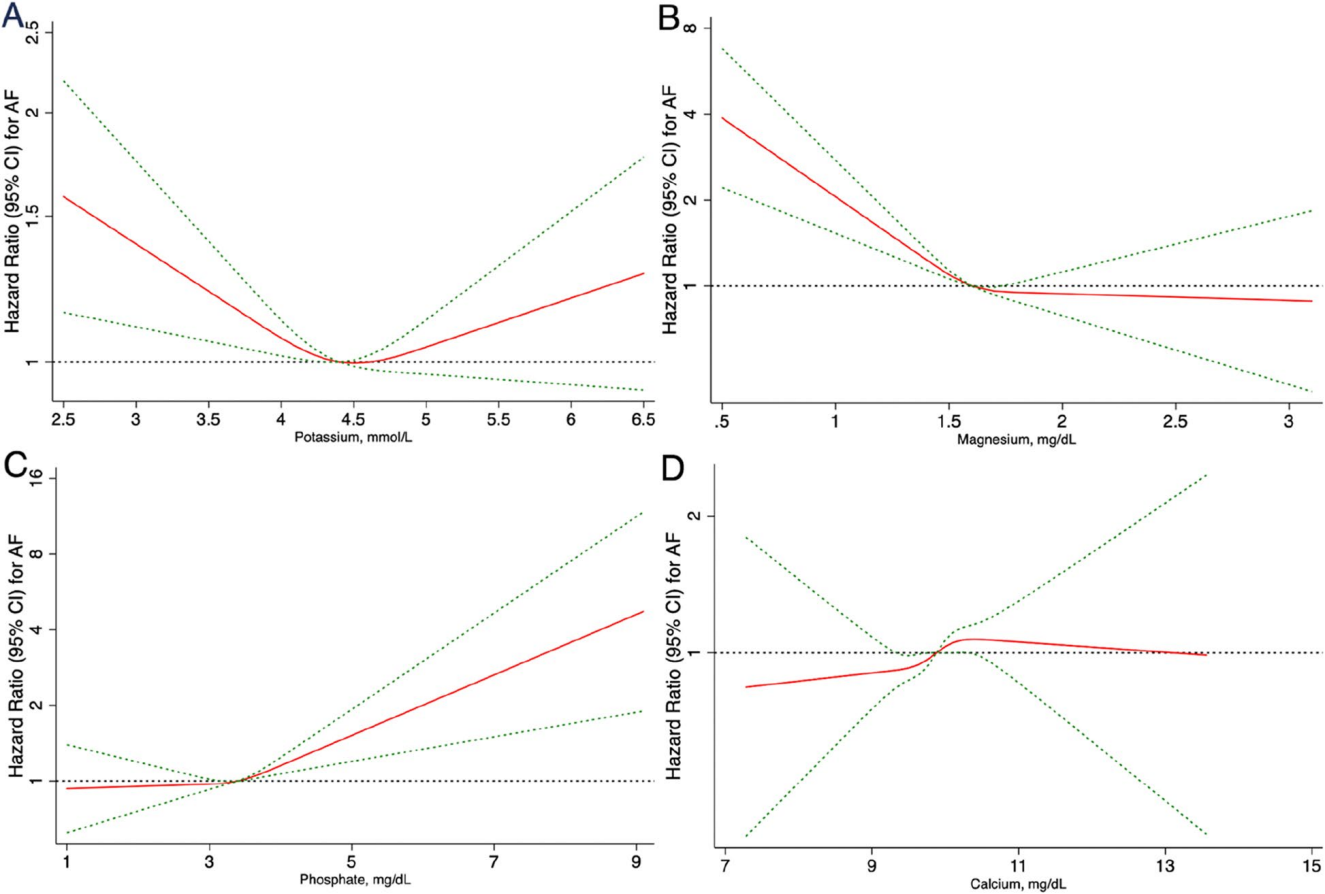
Dose-response relationship between change in serum electrolytes and AF.** A** The association between change in serum potassium with AF. Restricted cubic splines confirmed that serum potassium was inversely associated with risk of AF. **B** The association between change in serum magnesium with AF. Restricted cubic splines confirmed that serum magnesium was inversely associated with risk of AF. **C** The association between change in serum phosphate with AF. Restricted cubic spline presented a linear relation for associations between serum phosphate levels and AF risk and suggested a significant positive association. **D** The association between change in serum calcium with AF. There was no specific dose-response relationship between serum calcium and risk of AF. The curves are plotted using restricted cubic splines. The red curve represents hazard ratio, while the green curve represents the 95% confidence interval. AF, atrial fibrillation

The Kaplan-Meier survival curves in Fig. 2 showed similar results, while restricted cubic splines confirmed that serum potassium and magnesium levels were inversely associated with AF risk (Fig. 3). A linear relationship between serum phosphate levels and the AF risk was also presented by restricted cubic spline, indicating a significant positive association (Fig. 3). Nevertheless, there was no specific dose-response relationship between serum calcium and AF risk (Fig. 3).

### Mendelian randomization (MR) analysis

Summary information on the genetic variants and their associations with serum electrolytes and AF are presented in Supplemental Tables 1 to 4. In addition, details of studies and datasets used for MR analysis are also given in Supplemental Table 5. Variance (R^2^) in the MR study refers to the proportion of total variation in the exposure that is explained by the genetic instruments.

**Fig. 4:**
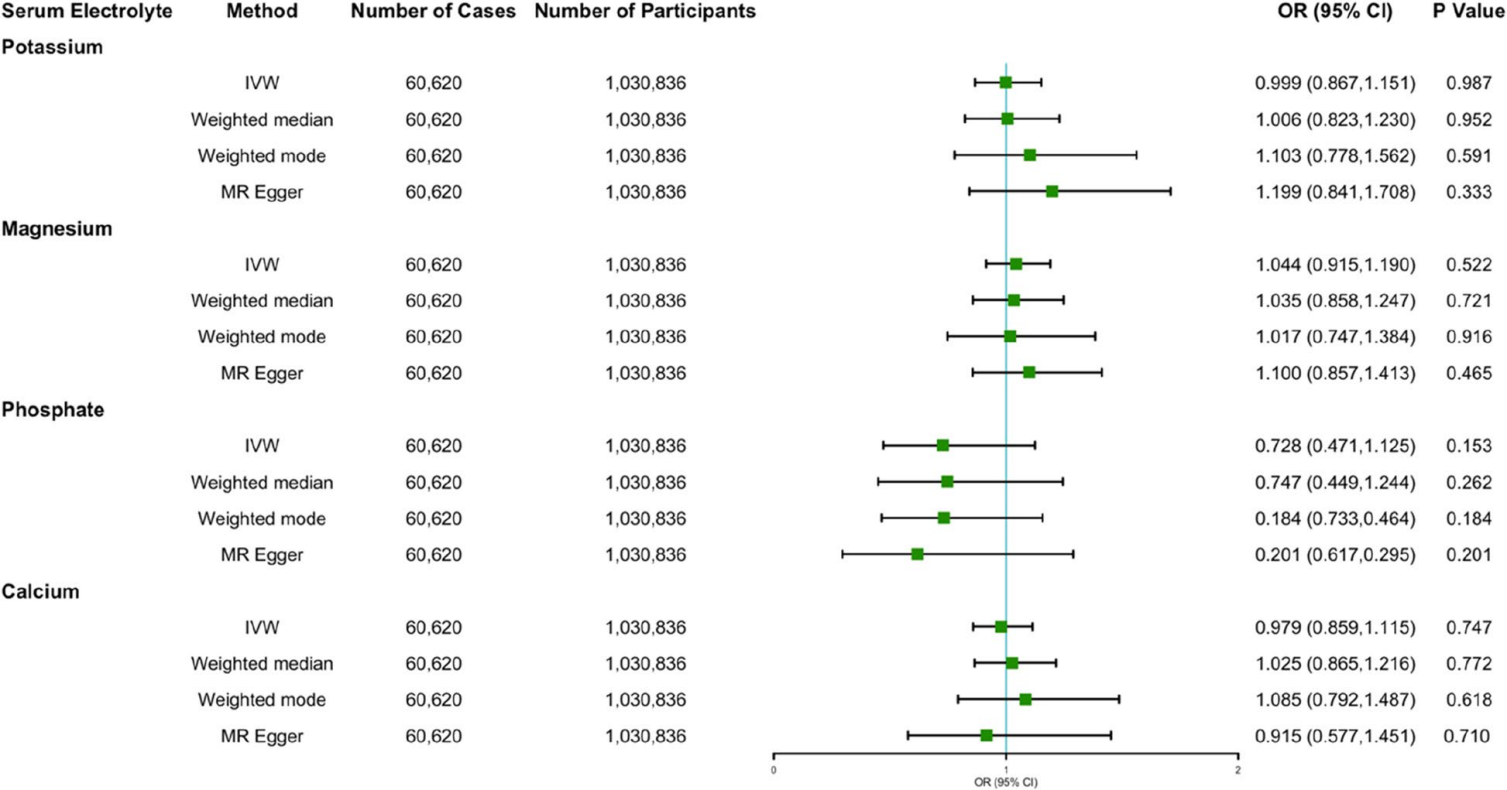
Causal association between serum electrolytes with AF. Effect of serum electrolytes on AF is consistent using different methods. IVW = inverse variance weighted

In the primary IVW analysis, no association was observed between genetically predicted circulating electrolyte concentration and AF risk, as shown in Table 3. The OR for AF was 0.999 (95% CI: 0.867 to 1.151) for serum potassium, while 1.044 (95% CI: 0.915 to 1.190) for serum magnesium, 0.728 (95% CI: 0.471 to 1.125) for serum phosphate, and 0.979 (95% CI: 0.859 to 1.115) for serum calcium, respectively. The forest plots of causal effects between electrolytes-associated SNPs and AF risk are displayed in Supplemental Fig. 1 to 4. Of note, the estimates were consistent in complementary analysis using weighted median, weighted mode, and MR Egger (Fig. 4; Table 3).

**Table 3:** Causal associations between genetically determined serum electrolytes and the risk of AF in Mendelian Randomization analyses

Exposure-outcome	Method	Causal estimate				
		SNP	OR	95% CI		*P* value
Potassium-AF	Inverse variance weighted	15	0.999	0.867	1.151	0.987
	Weighted median	15	1.006	0.823	1.230	0.952
	Weighted mode	15	1.103	0.778	1.562	0.591
	MR Egger	15	1.199	0.841	1.708	0.333
	Test for Heterogeneity: *P* = 0.781 (MR-Egger) and *P* = 0.754 (IVW)					
	Test for Horizontal pleiotropy: MR-Egger intercept = -0.0073, se = 0.00660, *P* = 0.290					
Magnesium-AF	Inverse variance weighted	19	1.044	0.915	1.190	0.522
	Weighted median	19	1.035	0.858	1.247	0.721
	Weighted mode	19	1.017	0.747	1.384	0.916
	MR Egger	19	1.100	0.857	1.413	0.465
	Test for Heterogeneity: *P* = 0.309 (MR-Egger) and *P* = 0.355 (IVW)					
	Test for Horizontal pleiotropy: MR-Egger intercept = -0.0026, se = 0.00536, *P* = 0.632					
Phosphate-AF	Inverse variance weighted	143	0.728	0.471	1.125	0.153
	Weighted median	143	0.747	0.449	1.244	0.262
	Weighted mode	143	0.733	0.464	1.157	0.184
	MR Egger	143	0.617	0.295	1.289	0.201
	Test for Heterogeneity: *P* = 1.751E-18 (MR-Egger) and *P* = 2.211E-18 (IVW)					
	Test for Horizontal pleiotropy: MR-Egger intercept = 0.0010, se = 0.00189, *P* = 0.586					
Calcium-AF	Inverse variance weighted	20	0.979	0.859	1.115	0.747
	Weighted median	20	1.025	0.865	1.216	0.772
	Weighted mode	20	1.085	0.792	1.487	0.618
	MR Egger	20	0.915	0.577	1.451	0.710
	Test for Heterogeneity: *P* = 0.186 (MR-Egger) and *P* = 0.227 (IVW)					
	Test for Horizontal pleiotropy: MR-Egger intercept = 0.0024, se = 0.00804, *P* = 0.768					

*AF* atrial fibrillation, *SNP* single-nucleotide polymorphism, *OR* odds ratio, *CI* confidence interval

In the present study, several sensitivity analyses were carried out to ensure the robustness of the findings. According to MR-Egger regression analysis, there was no indication of horizontal pleiotropy (MR-Egger for potassium, intercept = -0.0073, *p* = 0.290; MR-Egger for magnesium, intercept = -0.0026, *p* = 0.632; MR-Egger for phosphate, intercept = 0.0010, *p* = 0.586; MR-Egger for calcium, intercept = 0.0024, *p* = 0.768, Table 3, Supplemental Fig. 5 to 8). To test the sensitivity of the findings to specific variations, a leave-one-out analysis was performed, sequentially eliminating one SNP at a time, and results demonstrated that the likelihood of particular SNPs skewing the causal association was low (Supplemental Fig. 9 to 12). None of the funnel plots showed asymmetry (Supplemental Fig. 13 to 16).

No heterogeneity was detected in the Cochran Q test, except for investigating a causal relation between serum phosphate and AF (P for the MR-Egger = 1.751E-18; P for the IVW = 2.211E-18, Table 3). However, the MR estimate was still valid because the application of random-effect IVW in this study might balance the pooled heterogeneity. Furthermore, Egger the intercept revealed no pleiotropy, implying no pleiotropic bias was introduced into MR estimates in the setting of heterogeneity (Supplemental Fig. 7).

To summarize, the MR findings suggested that serum electrolytes were not causal determinants of AF risk (Fig. 4).

## Discussion

### Principal findings

The main findings of epidemiological analysis from the ARIC study can be summarized as follows: (i) both serum potassium and magnesium levels were inversely related to AF risk in an essentially linear pattern; (ii) higher serum phosphate levels were associated with a higher AF incident risk; (iii) no correlation was observed for AF risk and serum calcium levels. Moreover, using genetic analysis of large-scale summary GWAS data, MR results highlighted that genetically predicted serum electrolytes were not causally associated with AF risk. Consequently, serum electrolytes such as potassium, magnesium and phosphate may act as distinguished prognostic factors.

### Comparison with prior studies

Previous observational findings suggested that low potassium, magnesium, and high phosphate levels increase the risk of AF. Still, calcium levels were unrelated to AF [7–9]. Our observational study’s findings supported those of the studies described above. However, previous studies on the classification of electrolytes were too general to effectively clarify the association between various electrolyte concentrations and AF. Our study pinpointed the electrolyte concentrations that raise the incidence of AF. Crucially, these observational studies have yet to be able to determine definitively whether serum electrolytes and AF are casually linked, despite being aware of this limitation [7, 8, 31].

Previous MR studies have highlighted the causal relationship between cardiovascular risk factors such as lipoprotein(a), blood pressure level, BMI and incident AF [32–34]. However, electrolyte imbalance is a prevalent clinical complication, and its role in the etiology of AF is still challenging to understand. Furthermore, these observational and MR findings represent the most comprehensive and large-scale assessment of causal associations between serum electrolytes and AF.

### Potential explanations for findings

Several mechanisms potentially explain the role of serum electrolytes in AF since AF events are frequently detected in HF and CHD patients, where AF frequently coexist [35]. Both HF and CHD are major risk factors for the development of AF. Moreover, HF and CHD patients often suffer from hypokalemia and hypomagnesemia during treatment due to using drugs like diuretics [36, 37]. Similarly, chronic kidney disease (CKD) with concurrent hyperphosphatemia is another AF risk factor [38]. Of note, hypertension, diabetes mellitus, pulmonary embolism, and other disorders can all contribute to the onset and progression of AF. Electrolyte imbalance are common in patients with these disorders. Our findings indicate that electrolyte imbalance is not the cause of AF. The disorders that complicate with electrolyte imbalance and are also substantial risk factors for AF demand special attention and concern. Furthermore, reverse causation was not considered plausible explanation because serum electrolytes were measured at baseline and the majority of incident AF occurred after a median follow-up of 19.7 years.

### Study implications

Both clinical and public health practice can benefit from the findings of our study. Given that it is common for underlying disorders such as HF, CHD, and CKD to coexist with electrolyte imbalance, prevention of AF should focus on underlying diseases rather than electrolyte imbalance alone. Electrolytes including potassium, magnesium, and phosphate, can be excellent prognostic factors, prompting physicians to investigate and rectify the underlying disorders causing AF. Of significant clinical benefit is the implication of our findings, which sheds light on the role that electrolytes might play in preventing AF.

### Strengths and limitations of the study

The utilization of large sample sizes in the analysis enabled the performing sufficiently robust and comprehensive analysis for incident AF and well-powered GWAS to acquire genetic instruments for MR studies, which are the major strengths of this study. Besides, other strengths included the well-matched prospective design in observational analysis and the robustness of MR findings due to the absence of horizontal pleiotropy in sensitivity analysis.

This study also encountered certain limitations. First, it was unrealistic that all AF cases were identified in the ARIC study because AF can be paroxysmal and asymptomatic. Second, the observational study was limited to one baseline assessment of serum electrolytes. Given the limitations of the observational study, MR analysis was also carried out to make up for the shortcomings of the observational study. Third, limitations of the observational study include residual confounding, time-varying confounding, unmeasured confounding, potential for selection bias. Fourth, although no causal associations between electrolytes and AF risk were detected in the MR study, we could not completely rule out the possibility that the effect size is too small to be identified even within our large sample size. Fifth, given that GWAS data for AF were insufficient to calculate incidence, we are unable to provide specific data on the incidence of AF in MR. Last but not least, MR datasets comprised data from European ancestry, which limited the application of the findings to populations outside of European origin. Future experimental work and randomized controlled trials are required to elucidate further underlying physiological mechanisms and effective preventive measures for AF.

## Conclusion

Observational study implicated that hypokalemia, hypomagnesemia and hyperphosphatemia were associated with incident AF. However, MR analysis did not demonstrate that serum electrolytes play a causal role in the AF etiology. As a result, therapies aiming to prevent AF by treating electrolyte disorders such as hypokalemia, hypomagnesemia, and hyperphosphatemia are of limited clinical benefit.

### Supplementary Information

**Supplementary Material 1.**

## Data Availability

Data from the ARIC study was available to all researchers upon application. The used GWAS data were publicly available and their origins were described appropriately in the manuscript (https://gwas.mrcieu.ac.uk). The detailed information and codes required to reanalyze the data in this work are available from the corresponding authors upon reasonable request.
