# Supplementary material for: Serum electrolyte concentrations and risk of atrial fibrillation: an observational and mendelian randomization study
Source: BMC Genomics. 2024 Mar 16;25:280. doi: 10.1186/s12864-024-10197-2 (PMC10944597; doi:10.1186/s12864-024-10197-2)
Supplement: Supplementary file 1 — Supplementary Material 1. [file 12864_2024_10197_MOESM1_ESM.docx]

# **Supplementary files**

**Online Table 1** Characteristics of the SNPs associated with serum potassium and AF

**Online Table 2** Characteristics of the SNPs associated with serum magnesium and AF

**Online Table 3** Characteristics of the SNPs associated with serum calcium and AF

**Online Table 4** Characteristics of the SNPs associated with serum phosphate and AF

**Online Table 5** Details of studies and datasets used for MR analyses

**Online Figure 1** Forest plot of causal effects between potassium-associated SNPs and risk of AF.

**Online Figure 2** Forest plot of causal effects between magnesium-associated SNPs and risk of AF.

**Online Figure 3** Forest plot of causal effects between phosphate-associated SNPs and risk of AF.

**Online Figure 4** Forest plot of causal effects between calcium-associated SNPs and risk of AF.

**Online Figure 5** Scatter plot of causal effects between potassium-associated SNPs and risk of AF.

**Online Figure 6** Scatter plot of causal effects between magnesium-associated SNPs and risk of AF.

**Online Figure 7** Scatter plot of causal effects between phosphate-associated SNPs and risk of AF.

**Online Figure 8** Scatter plot of causal effects between calcium-associated SNPs and risk of AF.

**Online Figure 9** MR leave-one-out sensitivity analysis for serum potassium on AF.

**Online Figure 10** MR leave-one-out sensitivity analysis for serum magnesium on AF.

**Online Figure 11** MR leave-one-out sensitivity analysis for serum phosphate on AF.

**Online Figure 12** MR leave-one-out sensitivity analysis for serum calcium on AF.

**Online Figure 13** Funnel plot of causal association between serum potassium on AF.

**Online Figure 14** Funnel plot of causal association between serum magnesium on AF.

**Online Figure 15** Funnel plot of causal association between serum phosphate on AF.

**Online Figure 16** Funnel plot of causal association between serum calcium on AF.

**Table 1 Characteristics of the SNPs associated with serum potassium and AF**

| **Chromosome** | **SNP** | **EA** | **OA** | **EAF** | **R^2^** | **F-statistic** | **Associations with serum potassium** | | | |  | **Associations with AF** | | |
| --- | --- | --- | --- | --- | --- | --- | --- | --- | --- | --- | --- | --- | --- | --- |
|  |  |  |  |  |  |  | **Beta** | **se** | **P value** |  | | **Beta** | **se** | **P value** |
| 1 | rs2745938 | T | G | 0.647 | 3.09E-04 | 18.778 | 0.026 | 0.006 | 4.10E-06 |  | | 0.005 | 0.007 | 0.512 |
| 3 | rs3772928 | C | T | 0.575 | 3.56E-04 | 20.25 | -0.027 | 0.006 | 1.50E-06 |  | | -0.004 | 0.007 | 0.59 |
| 5 | rs114300683 | A | G | 0.012 | 3.14E-04 | 21.16 | -0.115 | 0.025 | 4.00E-06 |  | | 0.016 | 0.037 | 0.673 |
| 5 | rs145857065 | C | T | 0.03 | 4.01E-04 | 23.837 | -0.083 | 0.017 | 1.60E-06 |  | | 0.021 | 0.026 | 0.417 |
| 5 | rs148244439 | T | C | 0.052 | 3.20E-04 | 22.563 | 0.057 | 0.012 | 3.90E-06 |  | | 0.011 | 0.016 | 0.498 |
| 8 | rs35579431 | T | C | 0.266 | 3.28E-04 | 23.361 | 0.029 | 0.006 | 2.00E-06 |  | | -0.013 | 0.008 | 0.102 |
| 8 | rs77126457 | A | G | 0.014 | 3.04E-04 | 20.841 | 0.105 | 0.023 | 4.80E-06 |  | | 0.004 | 0.036 | 0.911 |
| 9 | rs7040926 | C | T | 0.065 | 3.16E-04 | 21.496 | -0.051 | 0.011 | 4.40E-06 |  | | -0.018 | 0.014 | 0.198 |
| 10 | rs10764330 | G | A | 0.699 | 3.30E-04 | 21.778 | 0.028 | 0.006 | 2.60E-06 |  | | -0.001 | 0.007 | 0.854 |
| 10 | rs12412051 | C | G | 0.034 | 3.50E-04 | 23.684 | 0.073 | 0.015 | 1.20E-06 |  | | 0.015 | 0.019 | 0.435 |
| 10 | rs1360913 | T | A | 0.577 | 3.83E-04 | 21.778 | 0.028 | 0.006 | 4.40E-07 |  | | -0.003 | 0.007 | 0.657 |
| 11 | rs361294 | C | A | 0.691 | 3.35E-04 | 21.778 | -0.028 | 0.006 | 2.00E-06 |  | | 0.007 | 0.007 | 0.321 |
| 11 | rs7479680 | C | A | 0.144 | 3.56E-04 | 22.563 | -0.038 | 0.008 | 9.00E-07 |  | | -0.0001 | 0.01 | 0.989 |
| 12 | rs12296227 | T | G | 0.267 | 3.07E-04 | 21.778 | 0.028 | 0.006 | 3.80E-06 |  | | -0.008 | 0.008 | 0.292 |
| 14 | rs77824658 | G | A | 0.09 | 3.17E-04 | 19.36 | 0.044 | 0.01 | 4.40E-06 |  | | 0.0003 | 0.012 | 0.981 |
| 16 | rs146963873 | A | T | 0.008 | 3.72E-04 | 21.496 | -0.153 | 0.033 | 4.00E-06 |  | | -0.053 | 0.052 | 0.311 |

AF, atrial fibrillation; SNP, single-nucleotide polymorphism; EA, effect allele; OA, other allele; EAF, effect allele frequency; se, standard error.

**Table 2 Characteristics of the SNPs associated with serum magnesium and AF**

| **Chromosome** | **SNP** | **EA** | **OA** | **EAF** | **R^2^** | **F-statistic** | **Associations with serum magnesium** | | | | |  | **Associations with AF** | | |
| --- | --- | --- | --- | --- | --- | --- | --- | --- | --- | --- | --- | --- | --- | --- | --- |
|  |  |  |  |  |  |  | **Beta** | **se** | **P value** |  | **Beta** | | | **se** | **P value** |
| 1 | rs2745938 | T | G | 0.647 | 3.33E-04 | 20.25 | 0.027 | 0.006 | 1.60E-06 |  | 0.005 | | | 0.007 | 0.512 |
| 2 | rs116740989 | A | C | 0.012 | 3.30E-04 | 22.278 | 0.118 | 0.025 | 2.80E-06 |  | -0.011 | | | 0.031 | 0.712 |
| 4 | rs114575778 | A | G | 0.01 | 4.16E-04 | 28.841 | -0.145 | 0.027 | 1.20E-07 |  | 0.027 | | | 0.04 | 0.493 |
| 4 | rs140205161 | G | A | 0.007 | 3.65E-04 | 21.424 | 0.162 | 0.035 | 4.10E-06 |  | 0.037 | | | 0.039 | 0.342 |
| 5 | rs116028267 | G | C | 0.038 | 3.28E-04 | 22.903 | -0.067 | 0.014 | 3.90E-06 |  | 0.002 | | | 0.02 | 0.926 |
| 5 | rs4535437 | G | A | 0.245 | 3.33E-04 | 25 | 0.03 | 0.006 | 1.20E-06 |  | 0.004 | | | 0.008 | 0.583 |
| 6 | rs114989460 | C | T | 0.018 | 3.26E-04 | 20.898 | 0.096 | 0.021 | 4.60E-06 |  | -0.012 | | | 0.031 | 0.69 |
| 7 | rs144862520 | T | C | 0.041 | 3.85E-04 | 21.778 | -0.07 | 0.015 | 4.70E-06 |  | -0.022 | | | 0.023 | 0.344 |
| 8 | rs77126457 | A | G | 0.014 | 3.65E-04 | 25 | 0.115 | 0.023 | 4.50E-07 |  | 0.004 | | | 0.036 | 0.911 |
| 9 | rs7022555 | T | C | 0.012 | 3.47E-04 | 23.426 | -0.121 | 0.025 | 1.80E-06 |  | -0.05 | | | 0.035 | 0.152 |
| 9 | rs76330086 | T | C | 0.015 | 3.07E-04 | 21.496 | -0.102 | 0.022 | 4.40E-06 |  | -0.045 | | | 0.034 | 0.179 |
| 10 | rs1247081 | T | G | 0.508 | 3.38E-04 | 27.04 | 0.026 | 0.005 | 1.70E-06 |  | 0.009 | | | 0.007 | 0.187 |
| 11 | rs116979507 | T | C | 0.039 | 3.07E-04 | 20.898 | 0.064 | 0.014 | 4.60E-06 |  | -0.02 | | | 0.02 | 0.315 |
| 11 | rs573905 | G | A | 0.547 | 3.89E-04 | 31.36 | 0.028 | 0.005 | 3.20E-07 |  | 0.001 | | | 0.007 | 0.926 |
| 12 | rs147150587 | A | G | 0.01 | 3.24E-04 | 20.898 | 0.128 | 0.028 | 3.40E-06 |  | -0.012 | | | 0.038 | 0.752 |
| 13 | rs7339029 | T | G | 0.134 | 3.01E-04 | 20.25 | 0.036 | 0.008 | 4.70E-06 |  | 0.01 | | | 0.01 | 0.319 |
| 16 | rs146963873 | A | T | 0.008 | 4.48E-04 | 25.917 | -0.168 | 0.033 | 3.60E-07 |  | -0.053 | | | 0.052 | 0.311 |
| 18 | rs1559583 | C | T | 0.785 | 3.04E-04 | 18.367 | -0.03 | 0.007 | 3.50E-06 |  | 0.022 | | | 0.008 | 0.006 |
| 20 | rs111419911 | G | A | 0.31 | 3.60E-04 | 23.361 | -0.029 | 0.006 | 6.30E-07 |  | 0.005 | | | 0.008 | 0.507 |

AF, atrial fibrillation; SNP, single-nucleotide polymorphism; EA, effect allele; OA, other allele; EAF, effect allele frequency; se, standard error.

**Table 3 Characteristics of the SNPs associated with serum calcium and AF**

| **Chromosome** | **SNP** | **EA** | **OA** | **EAF** | **R^2^** | **F-statistic** | **Associations with serum calcium** | | | |  | **Associations with AF** | | |
| --- | --- | --- | --- | --- | --- | --- | --- | --- | --- | --- | --- | --- | --- | --- |
|  |  |  |  |  |  |  | **Beta** | **se** | **P value** |  | | **Beta** | **se** | **P value** |
| 2 | rs12618785 | G | C | 0.399 | 3.50E-04 | 20.25 | 0.027 | 0.006 | 1.20E-06 |  | | -0.004 | 0.007 | 0.591 |
| 2 | rs4988235 | A | G | 0.723 | 4.10E-04 | 28.444 | 0.032 | 0.006 | 7.40E-08 |  | | 0.018 | 0.008 | 0.024 |
| 4 | rs73238581 | A | C | 0.347 | 3.30E-04 | 20.25 | -0.027 | 0.006 | 2.00E-06 |  | | 0.0004 | 0.007 | 0.96 |
| 5 | rs4535437 | G | A | 0.245 | 3.79E-04 | 28.444 | 0.032 | 0.006 | 4.50E-07 |  | | 0.004 | 0.008 | 0.583 |
| 5 | rs62347998 | T | C | 0.042 | 3.30E-04 | 20.898 | 0.064 | 0.014 | 3.40E-06 |  | | -0.001 | 0.019 | 0.975 |
| 7 | rs39308 | A | G | 0.265 | 3.51E-04 | 25 | 0.03 | 0.006 | 1.50E-06 |  | | 0.003 | 0.008 | 0.711 |
| 8 | rs1714800 | C | G | 0.662 | 3.51E-04 | 21.778 | 0.028 | 0.006 | 1.00E-06 |  | | -0.004 | 0.007 | 0.561 |
| 8 | rs2443773 | G | T | 0.506 | 3.64E-04 | 29.16 | 0.027 | 0.005 | 5.10E-07 |  | | 0.007 | 0.007 | 0.306 |
| 8 | rs7464794 | C | T | 0.499 | 3.38E-04 | 27.04 | -0.026 | 0.005 | 9.20E-07 |  | | 0.007 | 0.007 | 0.327 |
| 9 | rs117456360 | A | G | 0.043 | 3.27E-04 | 20.25 | 0.063 | 0.014 | 4.40E-06 |  | | -0.028 | 0.021 | 0.173 |
| 10 | rs1219820 | T | C | 0.029 | 3.25E-04 | 22.563 | -0.076 | 0.016 | 1.70E-06 |  | | -0.03 | 0.022 | 0.166 |
| 11 | rs11030416 | A | G | 0.042 | 3.40E-04 | 21.556 | -0.065 | 0.014 | 1.50E-06 |  | | -0.003 | 0.017 | 0.863 |
| 12 | rs7972943 | A | T | 0.066 | 5.70E-04 | 23.592 | -0.068 | 0.014 | 1.30E-06 |  | | 0.038 | 0.024 | 0.107 |
| 12 | rs7972943 | A | T | 0.066 | 5.70E-04 | 23.592 | -0.068 | 0.014 | 1.30E-06 |  | | -0.005 | 0.014 | 0.715 |
| 15 | rs753899 | C | T | 0.092 | 3.54E-04 | 26.123 | 0.046 | 0.009 | 7.40E-07 |  | | -0.021 | 0.012 | 0.069 |
| 16 | rs35683760 | G | A | 0.229 | 3.39E-04 | 26.694 | -0.031 | 0.006 | 1.50E-06 |  | | 0.013 | 0.008 | 0.105 |
| 17 | rs8067154 | A | G | 0.219 | 3.08E-04 | 18.367 | -0.03 | 0.007 | 4.60E-06 |  | | -0.006 | 0.008 | 0.479 |
| 18 | rs17712285 | G | A | 0.034 | 3.31E-04 | 22.404 | -0.071 | 0.015 | 1.70E-06 |  | | -0.016 | 0.024 | 0.508 |
| 19 | rs1974821 | A | G | 0.152 | 3.34E-04 | 20.25 | 0.036 | 0.008 | 2.30E-06 |  | | 0.002 | 0.01 | 0.852 |
| 19 | rs8109178 | T | A | 0.121 | 3.24E-04 | 23.766 | 0.039 | 0.008 | 3.00E-06 |  | | 0.007 | 0.012 | 0.537 |
| 21 | rs11088797 | A | G | 0.114 | 3.23E-04 | 25 | 0.04 | 0.008 | 3.10E-06 |  | | -0.013 | 0.011 | 0.212 |

AF, atrial fibrillation; SNP, single-nucleotide polymorphism; EA, effect allele; OA, other allele; EAF, effect allele frequency; se, standard error.

**Table 4 Characteristics of the SNPs associated with serum phosphate and AF**

| **Chromosome** | **SNP** | **EA** | **OA** | **EAF** | **R^2^** | **F-statistic** | **Associations with serum phosphate** | | | |  | **Associations with AF** | | |
| --- | --- | --- | --- | --- | --- | --- | --- | --- | --- | --- | --- | --- | --- | --- |
|  |  |  |  |  |  |  | **Beta** | **se** | **P value** |  | | **Beta** | **se** | **P value** |
| 1 | rs113487566 | T | C | 0.025 | 4.88E-06 | 59.172 | -0.01 | 0.0013 | 5.87E-16 |  | | -0.015 | 0.025 | 0.5444 |
| 1 | rs11801716 | T | C | 0.046 | 2.19E-06 | 30.864 | -0.005 | 0.0009 | 9.47E-09 |  | | -0.0051 | 0.015 | 0.7307 |
| 1 | rs12044944 | T | C | 0.193 | 2.80E-06 | 36 | 0.003 | 0.0005 | 4.29E-09 |  | | 0.0015 | 0.009 | 0.8559 |
| 1 | rs12059422 | C | T | 0.023 | 1.01E-05 | 133.136 | 0.015 | 0.0013 | 3.32E-29 |  | | 0.0429 | 0.022 | 0.0466101 |
| 1 | rs12132412 | G | A | 0.388 | 1.37E-04 | 1806.25 | 0.017 | 0.0004 | 1.00E-200 |  | | -0.0109 | 0.007 | 0.1248 |
| 1 | rs12409158 | C | G | 0.032 | 2.23E-06 | 29.752 | -0.006 | 0.0011 | 1.70E-08 |  | | -0.0364 | 0.024 | 0.1261 |
| 1 | rs12727188 | G | T | 0.089 | 4.05E-06 | 51.02 | -0.005 | 0.0007 | 6.82E-15 |  | | 0.0076 | 0.011 | 0.4965 |
| 1 | rs4072537 | C | T | 0.681 | 1.74E-06 | 25 | -0.002 | 0.0004 | 3.95E-08 |  | | 9.00E-04 | 0.008 | 0.9143 |
| 1 | rs71641333 | A | T | 0.075 | 3.47E-06 | 39.063 | -0.005 | 0.0008 | 2.73E-09 |  | | 0.0381 | 0.014 | 0.00690399 |
| 1 | rs72660359 | T | G | 0.037 | 1.60E-05 | 225 | 0.015 | 0.001 | 1.30E-45 |  | | 0.0217 | 0.018 | 0.2326 |
| 1 | rs7529925 | T | C | 0.738 | 3.48E-06 | 56.25 | 0.003 | 0.0004 | 7.01E-12 |  | | -0.0047 | 0.008 | 0.5318 |
| 1 | rs7555660 | T | C | 0.085 | 1.26E-05 | 165.306 | 0.009 | 0.0007 | 4.91E-38 |  | | 0.0149 | 0.012 | 0.226 |
| 1 | rs79472736 | A | G | 0.035 | 3.31E-06 | 40.496 | -0.007 | 0.0011 | 3.45E-11 |  | | 0.0194 | 0.021 | 0.3537 |
| 2 | rs11684211 | A | G | 0.353 | 4.11E-06 | 56.25 | 0.003 | 0.0004 | 3.81E-10 |  | | -0.0069 | 0.007 | 0.3216 |
| 2 | rs2303917 | A | G | 0.108 | 3.08E-06 | 44.444 | -0.004 | 0.0006 | 6.18E-09 |  | | -0.0254 | 0.011 | 0.0162099 |
| 2 | rs55923451 | A | G | 0.179 | 2.65E-06 | 36 | -0.003 | 0.0005 | 5.39E-11 |  | | 0.0044 | 0.009 | 0.615399 |
| 2 | rs6751217 | T | C | 0.632 | 4.19E-06 | 56.25 | 0.003 | 0.0004 | 6.03E-13 |  | | -0.0112 | 0.007 | 0.1023 |
| 2 | rs72974112 | T | C | 0.237 | 3.25E-06 | 36 | -0.003 | 0.0005 | 4.78E-10 |  | | 0.0035 | 0.008 | 0.6531 |
| 2 | rs77149268 | T | C | 0.105 | 9.21E-06 | 136.111 | 0.007 | 0.0006 | 5.82E-25 |  | | -0.0206 | 0.011 | 0.0648007 |
| 2 | rs838717 | A | G | 0.566 | 1.23E-05 | 156.25 | 0.005 | 0.0004 | 3.04E-35 |  | | -0.0042 | 0.007 | 0.5332 |
| 2 | rs878919 | C | T | 0.397 | 4.31E-06 | 56.25 | 0.003 | 0.0004 | 3.10E-15 |  | | -0.012 | 0.007 | 0.0774194 |
| 2 | rs896788 | T | C | 0.155 | 4.19E-06 | 64 | 0.004 | 0.0005 | 6.52E-15 |  | | 0.0107 | 0.009 | 0.2332 |
| 3 | rs11705850 | C | T | 0.079 | 2.33E-06 | 32.653 | -0.004 | 0.0007 | 9.86E-10 |  | | 0.0093 | 0.012 | 0.4401 |
| 3 | rs1483842 | C | T | 0.481 | 4.49E-06 | 56.25 | -0.003 | 0.0004 | 1.42E-12 |  | | -7.00E-04 | 0.007 | 0.9187 |
| 3 | rs17036160 | T | C | 0.118 | 3.33E-06 | 44.444 | -0.004 | 0.0006 | 5.67E-11 |  | | 0.0344 | 0.01 | 0.0008104 |
| 3 | rs4855880 | A | G | 0.516 | 2.00E-06 | 25 | -0.002 | 0.0004 | 4.18E-08 |  | | -0.0054 | 0.011 | 0.6148 |
| 3 | rs56328339 | T | C | 0.163 | 6.82E-06 | 100 | 0.005 | 0.0005 | 1.05E-22 |  | | 0.0034 | 0.009 | 0.7134 |
| 3 | rs6780593 | A | G | 0.153 | 6.48E-06 | 100 | -0.005 | 0.0005 | 8.55E-17 |  | | -0.001 | 0.009 | 0.9137 |
| 3 | rs73186030 | T | C | 0.128 | 3.21E-05 | 400 | -0.012 | 0.0006 | 5.39E-87 |  | | 0.0167 | 0.01 | 0.0965295 |
| 3 | rs7625643 | G | A | 0.448 | 4.45E-06 | 56.25 | 0.003 | 0.0004 | 7.39E-11 |  | | 0.0338 | 0.01 | 0.00085049 |
| 4 | rs10013062 | G | A | 0.352 | 1.82E-06 | 25 | 0.002 | 0.0004 | 3.46E-08 |  | | 0.0313 | 0.007 | 7.25E-06 |
| 4 | rs4690098 | T | C | 0.235 | 3.24E-06 | 36 | -0.003 | 0.0005 | 2.08E-08 |  | | 0.0067 | 0.008 | 0.3924 |
| 4 | rs62289280 | C | T | 0.305 | 3.82E-06 | 56.25 | -0.003 | 0.0004 | 1.09E-11 |  | | -0.0163 | 0.007 | 0.0253998 |
| 4 | rs6841258 | T | C | 0.166 | 2.49E-06 | 36 | 0.003 | 0.0005 | 5.29E-09 |  | | -9.00E-04 | 0.009 | 0.9214 |
| 4 | rs7672820 | C | G | 0.186 | 4.84E-06 | 64 | 0.004 | 0.0005 | 6.26E-18 |  | | -0.0029 | 0.009 | 0.737199 |
| 5 | rs10051765 | C | T | 0.332 | 2.84E-05 | 400 | -0.008 | 0.0004 | 3.03E-87 |  | | 0.0041 | 0.007 | 0.564999 |
| 5 | rs10900829 | G | A | 0.582 | 4.38E-06 | 56.25 | 0.003 | 0.0004 | 1.92E-13 |  | | -0.0104 | 0.007 | 0.1261 |
| 5 | rs12518871 | T | C | 0.41 | 4.35E-06 | 56.25 | -0.003 | 0.0004 | 2.57E-10 |  | | 0.0025 | 0.007 | 0.7142 |
| 5 | rs1432679 | T | C | 0.553 | 1.98E-06 | 25 | 0.002 | 0.0004 | 1.62E-09 |  | | -0.0123 | 0.007 | 0.0654305 |
| 5 | rs62383568 | C | T | 0.35 | 1.82E-06 | 25 | -0.002 | 0.0004 | 3.35E-09 |  | | 0.0142 | 0.007 | 0.0463404 |
| 5 | rs706300 | G | A | 0.858 | 6.09E-06 | 69.444 | -0.005 | 0.0006 | 2.96E-22 |  | | 0.0108 | 0.01 | 0.2636 |
| 5 | rs7705189 | G | A | 0.467 | 1.99E-06 | 25 | -0.002 | 0.0004 | 4.64E-10 |  | | -0.0085 | 0.007 | 0.2053 |
| 5 | rs7726961 | T | C | 0.426 | 1.96E-06 | 25 | -0.002 | 0.0004 | 6.85E-09 |  | | -0.0164 | 0.007 | 0.0149001 |
| 6 | rs13208790 | C | T | 0.651 | 1.82E-06 | 25 | 0.002 | 0.0004 | 9.66E-09 |  | | 0.0175 | 0.007 | 0.0134199 |
| 6 | rs145330264 | C | A | 0.005 | 1.02E-05 | 130.612 | -0.032 | 0.0028 | 5.75E-30 |  | | 0.0073 | 0.02 | 0.715201 |
| 6 | rs145330264 | C | A | 0.005 | 1.02E-05 | 130.612 | -0.032 | 0.0028 | 5.75E-30 |  | | 0.0307 | 0.077 | 0.690799 |
| 6 | rs17288460 | A | G | 0.18 | 2.66E-06 | 36 | -0.003 | 0.0005 | 7.84E-11 |  | | 0.0201 | 0.01 | 0.0360296 |
| 6 | rs1889208 | A | G | 0.336 | 4.02E-06 | 56.25 | 0.003 | 0.0004 | 3.76E-15 |  | | -0.0115 | 0.007 | 0.1043 |
| 6 | rs2982572 | T | C | 0.421 | 7.80E-06 | 100 | -0.004 | 0.0004 | 1.83E-25 |  | | -0.0239 | 0.007 | 0.0003747 |
| 6 | rs3813498 | T | C | 0.813 | 7.60E-06 | 100 | 0.005 | 0.0005 | 3.42E-24 |  | | 0.0013 | 0.009 | 0.8785 |
| 6 | rs453639 | A | C | 0.641 | 5.57E-05 | 756.25 | -0.011 | 0.0004 | 2.10E-150 |  | | 0.0116 | 0.007 | 0.1065 |
| 6 | rs4706554 | A | G | 0.476 | 4.49E-06 | 56.25 | 0.003 | 0.0004 | 2.22E-13 |  | | -0.0132 | 0.007 | 0.0474395 |
| 6 | rs4709746 | T | C | 0.134 | 2.09E-06 | 25 | 0.003 | 0.0006 | 1.97E-09 |  | | 0.0179 | 0.01 | 0.0698602 |
| 6 | rs4869745 | T | C | 0.287 | 3.68E-06 | 56.25 | 0.003 | 0.0004 | 6.73E-13 |  | | 0.0125 | 0.007 | 0.0900803 |
| 6 | rs56078331 | T | C | 0.054 | 6.54E-06 | 79.012 | 0.008 | 0.0009 | 2.07E-22 |  | | -0.0226 | 0.015 | 0.1359 |
| 6 | rs60447213 | T | G | 0.07 | 1.17E-04 | 1406.25 | -0.03 | 0.0008 | 1.00E-200 |  | | -0.0118 | 0.013 | 0.3467 |
| 6 | rs6911602 | A | G | 0.132 | 1.47E-05 | 177.778 | 0.008 | 0.0006 | 5.07E-46 |  | | 0.0145 | 0.01 | 0.1307 |
| 6 | rs74551305 | C | T | 0.056 | 5.18E-06 | 60.494 | 0.007 | 0.0009 | 1.23E-16 |  | | -0.0266 | 0.031 | 0.3847 |
| 6 | rs805288 | T | C | 0.249 | 1.50E-06 | 16 | 0.002 | 0.0005 | 4.98E-08 |  | | -0.0203 | 0.008 | 0.0117101 |
| 6 | rs912981 | C | A | 0.581 | 4.38E-06 | 56.25 | -0.003 | 0.0004 | 3.22E-11 |  | | 0.0067 | 0.007 | 0.3181 |
| 6 | rs9388490 | T | C | 0.437 | 4.43E-06 | 56.25 | 0.003 | 0.0004 | 4.59E-12 |  | | -4.00E-04 | 0.007 | 0.9498 |
| 6 | rs9689096 | C | A | 0.064 | 5.87E-06 | 76.563 | -0.007 | 0.0008 | 4.31E-18 |  | | 0.061 | 0.015 | 3.60E-05 |
| 7 | rs10279171 | T | A | 0.794 | 2.94E-06 | 36 | 0.003 | 0.0005 | 2.04E-09 |  | | 0.0085 | 0.008 | 0.3006 |
| 7 | rs10282449 | A | T | 0.166 | 4.43E-06 | 64 | 0.004 | 0.0005 | 2.15E-12 |  | | 0.0055 | 0.009 | 0.5348 |
| 7 | rs13232861 | G | A | 0.812 | 2.75E-06 | 36 | 0.003 | 0.0005 | 3.76E-10 |  | | -0.0059 | 0.009 | 0.4867 |
| 7 | rs144214505 | T | C | 0.042 | 2.90E-06 | 36 | -0.006 | 0.001 | 7.05E-09 |  | | -0.0068 | 0.019 | 0.726001 |
| 7 | rs2177470 | A | T | 0.682 | 3.90E-06 | 56.25 | -0.003 | 0.0004 | 2.60E-10 |  | | 0.0116 | 0.007 | 0.1132 |
| 7 | rs2460421 | T | A | 0.502 | 2.00E-06 | 25 | -0.002 | 0.0004 | 2.85E-08 |  | | -0.0211 | 0.007 | 0.00156599 |
| 7 | rs3757677 | G | A | 0.372 | 4.21E-06 | 56.25 | 0.003 | 0.0004 | 6.84E-14 |  | | -0.0088 | 0.007 | 0.2083 |
| 7 | rs4410790 | C | T | 0.634 | 4.18E-06 | 56.25 | 0.003 | 0.0004 | 2.41E-11 |  | | 0.0095 | 0.007 | 0.1699 |
| 8 | rs13273161 | T | A | 0.318 | 3.90E-06 | 56.25 | 0.003 | 0.0004 | 9.94E-13 |  | | -0.0181 | 0.007 | 0.01243 |
| 8 | rs2941483 | G | A | 0.571 | 4.41E-06 | 56.25 | 0.003 | 0.0004 | 1.27E-13 |  | | -0.0011 | 0.007 | 0.8707 |
| 8 | rs6983239 | T | G | 0.219 | 5.47E-06 | 64 | 0.004 | 0.0005 | 3.52E-16 |  | | -0.0094 | 0.008 | 0.2437 |
| 8 | rs7017252 | T | C | 0.383 | 1.89E-06 | 25 | 0.002 | 0.0004 | 3.53E-08 |  | | 0.0111 | 0.007 | 0.1058 |
| 9 | rs11144134 | C | T | 0.085 | 2.49E-06 | 32.653 | 0.004 | 0.0007 | 7.16E-10 |  | | 0.0035 | 0.013 | 0.782499 |
| 9 | rs28542318 | T | A | 0.834 | 2.49E-06 | 36 | 0.003 | 0.0005 | 1.75E-09 |  | | -0.0061 | 0.01 | 0.546 |
| 9 | rs296847 | T | G | 0.664 | 1.78E-06 | 25 | 0.002 | 0.0004 | 8.87E-09 |  | | 3.00E-04 | 0.007 | 0.9632 |
| 9 | rs7033278 | T | C | 0.293 | 3.73E-06 | 56.25 | 0.003 | 0.0004 | 5.96E-16 |  | | 7.00E-04 | 0.007 | 0.9235 |
| 9 | rs72716022 | T | C | 0.202 | 2.90E-06 | 36 | -0.003 | 0.0005 | 1.09E-10 |  | | -0.0098 | 0.009 | 0.2587 |
| 9 | rs7860558 | C | T | 0.701 | 3.77E-06 | 56.25 | -0.003 | 0.0004 | 3.13E-11 |  | | -7.00E-04 | 0.007 | 0.9204 |
| 10 | rs11191643 | G | C | 0.438 | 4.43E-06 | 56.25 | 0.003 | 0.0004 | 1.76E-12 |  | | 0.0129 | 0.007 | 0.05433 |
| 10 | rs12770024 | G | C | 0.194 | 5.00E-06 | 64 | 0.004 | 0.0005 | 4.92E-14 |  | | 0.002 | 0.009 | 0.8129 |
| 10 | rs2420055 | C | T | 0.843 | 4.24E-06 | 64 | -0.004 | 0.0005 | 4.21E-11 |  | | 0.0047 | 0.009 | 0.6095 |
| 10 | rs4509682 | A | G | 0.392 | 4.29E-06 | 56.25 | -0.003 | 0.0004 | 5.48E-15 |  | | -0.0307 | 0.007 | 6.29E-06 |
| 10 | rs485411 | C | T | 0.748 | 3.39E-06 | 36 | 0.003 | 0.0005 | 6.17E-13 |  | | -0.0035 | 0.008 | 0.649101 |
| 10 | rs6650130 | T | A | 0.713 | 3.68E-06 | 56.25 | 0.003 | 0.0004 | 2.78E-13 |  | | -0.0115 | 0.007 | 0.1144 |
| 10 | rs77282753 | C | T | 0.048 | 4.48E-06 | 60.494 | -0.007 | 0.0009 | 4.06E-14 |  | | -0.0067 | 0.015 | 0.6586 |
| 11 | rs10896012 | C | T | 0.218 | 5.46E-06 | 64 | -0.004 | 0.0005 | 6.47E-18 |  | | -0.0152 | 0.008 | 0.0688002 |
| 11 | rs11021221 | A | T | 0.171 | 2.55E-06 | 36 | -0.003 | 0.0005 | 2.18E-08 |  | | -0.0044 | 0.009 | 0.6255 |
| 11 | rs273587 | T | A | 0.678 | 3.93E-06 | 56.25 | -0.003 | 0.0004 | 7.53E-13 |  | | -0.0156 | 0.007 | 0.0293799 |
| 11 | rs2959652 | T | G | 0.908 | 2.67E-06 | 32.653 | -0.004 | 0.0007 | 3.13E-08 |  | | 0.0033 | 0.011 | 0.7596 |
| 11 | rs4938642 | C | G | 0.074 | 2.19E-06 | 32.653 | 0.004 | 0.0007 | 4.74E-08 |  | | 0.0086 | 0.014 | 0.5286 |
| 11 | rs61870274 | A | C | 0.078 | 5.18E-06 | 73.469 | 0.006 | 0.0007 | 7.92E-16 |  | | 0.0114 | 0.012 | 0.3467 |
| 11 | rs73632745 | T | C | 0.074 | 2.19E-06 | 32.653 | 0.004 | 0.0007 | 3.37E-08 |  | | 0.0025 | 0.013 | 0.8398 |
| 12 | rs10743976 | C | A | 0.851 | 1.24E-05 | 196 | 0.007 | 0.0005 | 7.00E-34 |  | | -0.0011 | 0.01 | 0.9092 |
| 12 | rs138278885 | CA | C | 0.101 | 6.54E-06 | 73.469 | -0.006 | 0.0007 | 3.68E-18 |  | | -0.0041 | 0.022 | 0.8494 |
| 12 | rs145978363 | G | A | 0.03 | 2.85E-06 | 34.028 | -0.007 | 0.0012 | 1.89E-09 |  | | 0.0186 | 0.022 | 0.3986 |
| 12 | rs1468908 | G | A | 0.492 | 1.25E-05 | 156.25 | 0.005 | 0.0004 | 6.28E-37 |  | | -0.0202 | 0.007 | 0.00236597 |
| 12 | rs17884869 | A | G | 0.025 | 3.12E-06 | 37.87 | 0.008 | 0.0013 | 2.58E-10 |  | | -0.0328 | 0.021 | 0.1134 |
| 12 | rs2970818 | A | T | 0.104 | 8.22E-05 | 1225 | 0.021 | 0.0006 | 1.00E-200 |  | | -0.0053 | 0.011 | 0.625101 |
| 12 | rs35026779 | T | C | 0.1 | 2.88E-06 | 32.653 | -0.004 | 0.0007 | 9.54E-11 |  | | 0.0266 | 0.011 | 0.02016 |
| 12 | rs4759844 | G | A | 0.351 | 7.29E-06 | 100 | 0.004 | 0.0004 | 1.22E-20 |  | | -0.0128 | 0.007 | 0.0679501 |
| 12 | rs596940 | C | T | 0.072 | 3.34E-06 | 39.063 | 0.005 | 0.0008 | 1.16E-11 |  | | 0.0071 | 0.013 | 0.581599 |
| 12 | rs61909254 | C | T | 0.86 | 2.41E-05 | 277.778 | 0.01 | 0.0006 | 8.49E-72 |  | | 0.0018 | 0.01 | 0.8519 |
| 12 | rs653178 | T | C | 0.517 | 7.99E-06 | 100 | -0.004 | 0.0004 | 3.60E-24 |  | | -0.0033 | 0.007 | 0.6256 |
| 13 | rs1570603 | A | G | 0.572 | 1.96E-06 | 25 | -0.002 | 0.0004 | 3.69E-08 |  | | 0.0091 | 0.007 | 0.176 |
| 13 | rs7324259 | T | G | 0.126 | 3.52E-06 | 44.444 | 0.004 | 0.0006 | 4.73E-10 |  | | 0.0043 | 0.011 | 0.684499 |
| 13 | rs9574586 | C | T | 0.331 | 1.77E-06 | 25 | -0.002 | 0.0004 | 2.35E-08 |  | | 7.00E-04 | 0.007 | 0.9158 |
| 14 | rs1286070 | C | T | 0.172 | 4.56E-06 | 64 | -0.004 | 0.0005 | 2.43E-15 |  | | 0.0036 | 0.009 | 0.6815 |
| 14 | rs4982711 | G | T | 0.571 | 1.96E-06 | 25 | 0.002 | 0.0004 | 1.05E-08 |  | | -0.0025 | 0.009 | 0.781601 |
| 14 | rs71413981 | A | G | 0.164 | 2.47E-06 | 36 | -0.003 | 0.0005 | 6.93E-09 |  | | 0.0134 | 0.01 | 0.1618 |
| 15 | rs12902894 | C | A | 0.458 | 7.94E-06 | 100 | 0.004 | 0.0004 | 8.14E-29 |  | | 0.002 | 0.007 | 0.764599 |
| 15 | rs339969 | A | C | 0.615 | 4.26E-06 | 56.25 | 0.003 | 0.0004 | 2.35E-11 |  | | 0.0099 | 0.007 | 0.1508 |
| 15 | rs34933034 | A | G | 0.153 | 9.33E-06 | 144 | -0.006 | 0.0005 | 7.19E-26 |  | | 3.00E-04 | 0.01 | 0.9726 |
| 16 | rs11646780 | A | T | 0.584 | 4.37E-06 | 56.25 | -0.003 | 0.0004 | 1.09E-10 |  | | 0.0104 | 0.007 | 0.1262 |
| 16 | rs2230742 | G | A | 0.846 | 4.17E-06 | 64 | -0.004 | 0.0005 | 6.74E-14 |  | | 0.0051 | 0.009 | 0.583399 |
| 16 | rs3213473 | A | T | 0.034 | 1.11E-05 | 139.669 | 0.013 | 0.0011 | 6.75E-31 |  | | -0.0204 | 0.026 | 0.4376 |
| 16 | rs3213473 | A | T | 0.034 | 1.11E-05 | 139.669 | 0.013 | 0.0011 | 6.75E-31 |  | | 0.0118 | 0.01 | 0.2553 |
| 16 | rs4077450 | T | G | 0.828 | 4.56E-06 | 64 | -0.004 | 0.0005 | 1.15E-15 |  | | -0.0133 | 0.009 | 0.1287 |
| 16 | rs41278174 | A | G | 0.027 | 1.52E-05 | 200.694 | 0.017 | 0.0012 | 1.31E-45 |  | | -0.0116 | 0.024 | 0.6231 |
| 16 | rs76819459 | G | A | 0.327 | 1.10E-05 | 156.25 | -0.005 | 0.0004 | 1.15E-34 |  | | -0.0045 | 0.007 | 0.5261 |
| 16 | rs9745989 | C | T | 0.628 | 4.21E-06 | 56.25 | 0.003 | 0.0004 | 9.31E-10 |  | | 0.0114 | 0.007 | 0.1237 |
| 17 | rs11078597 | C | T | 0.186 | 1.09E-05 | 144 | 0.006 | 0.0005 | 4.83E-28 |  | | -0.0135 | 0.009 | 0.1183 |
| 17 | rs111723017 | C | G | 0.088 | 4.01E-06 | 51.02 | 0.005 | 0.0007 | 1.89E-14 |  | | -0.0109 | 0.011 | 0.3179 |
| 17 | rs12941512 | T | C | 0.442 | 4.44E-06 | 56.25 | -0.003 | 0.0004 | 2.07E-16 |  | | 0.0047 | 0.007 | 0.4865 |
| 17 | rs2120222 | C | A | 0.148 | 2.04E-05 | 324 | -0.009 | 0.0005 | 9.73E-63 |  | | 0.0189 | 0.009 | 0.0385798 |
| 17 | rs2909212 | G | A | 0.297 | 1.67E-06 | 25 | 0.002 | 0.0004 | 2.31E-08 |  | | 0.0011 | 0.007 | 0.8768 |
| 17 | rs2955382 | T | C | 0.612 | 4.27E-06 | 56.25 | -0.003 | 0.0004 | 4.82E-15 |  | | -0.0138 | 0.007 | 0.0422503 |
| 17 | rs4795607 | T | C | 0.614 | 1.90E-06 | 25 | 0.002 | 0.0004 | 1.58E-09 |  | | 0.0023 | 0.007 | 0.7362 |
| 17 | rs5435 | C | T | 0.612 | 1.90E-06 | 25 | -0.002 | 0.0004 | 4.08E-08 |  | | -0.0101 | 0.007 | 0.1461 |
| 17 | rs55938136 | G | A | 0.226 | 5.60E-06 | 64 | -0.004 | 0.0005 | 6.77E-21 |  | | 0.0469 | 0.009 | 7.79E-08 |
| 17 | rs71368113 | G | C | 0.071 | 2.11E-06 | 25 | -0.004 | 0.0008 | 4.44E-08 |  | | -0.0178 | 0.015 | 0.2468 |
| 17 | rs76527351 | G | A | 0.226 | 5.60E-06 | 64 | -0.004 | 0.0005 | 7.59E-21 |  | | 0.0647 | 0.022 | 0.00317497 |
| 17 | rs8072297 | T | A | 0.231 | 1.28E-05 | 144 | -0.006 | 0.0005 | 3.29E-33 |  | | 0.0214 | 0.013 | 0.0870703 |
| 17 | rs8075449 | T | C | 0.259 | 3.45E-06 | 56.25 | -0.003 | 0.0004 | 5.03E-10 |  | | -0.0025 | 0.008 | 0.7406 |
| 17 | rs872386 | A | G | 0.531 | 7.97E-06 | 100 | -0.004 | 0.0004 | 2.60E-23 |  | | 0.025 | 0.007 | 0.0001875 |
| 18 | rs3897629 | A | T | 0.496 | 2.00E-06 | 25 | -0.002 | 0.0004 | 4.76E-10 |  | | -0.0041 | 0.007 | 0.5413 |
| 19 | rs10401230 | G | A | 0.494 | 2.00E-06 | 25 | -0.002 | 0.0004 | 9.47E-09 |  | | 0.0021 | 0.007 | 0.7643 |
| 19 | rs10415758 | T | A | 0.422 | 4.39E-06 | 56.25 | -0.003 | 0.0004 | 5.15E-11 |  | | -0.0044 | 0.007 | 0.5432 |
| 19 | rs12710029 | C | T | 0.776 | 3.13E-06 | 36 | -0.003 | 0.0005 | 7.06E-10 |  | | -0.0018 | 0.008 | 0.8239 |
| 19 | rs12985272 | A | G | 0.049 | 3.36E-06 | 44.444 | 0.006 | 0.0009 | 4.69E-12 |  | | 0.014 | 0.017 | 0.4026 |
| 19 | rs139796809 | T | C | 0.557 | 4.44E-06 | 56.25 | -0.003 | 0.0004 | 1.53E-11 |  | | -0.0056 | 0.013 | 0.6629 |
| 19 | rs308032 | T | G | 0.237 | 3.25E-06 | 36 | -0.003 | 0.0005 | 1.03E-09 |  | | 0.0027 | 0.009 | 0.7493 |
| 19 | rs78030362 | G | A | 0.074 | 4.93E-06 | 56.25 | 0.006 | 0.0008 | 6.50E-17 |  | | 0.0055 | 0.014 | 0.6924 |
| 19 | rs8105161 | C | T | 0.155 | 4.19E-06 | 44.444 | -0.004 | 0.0006 | 1.14E-10 |  | | -0.0032 | 0.01 | 0.742299 |
| 20 | rs17265513 | C | T | 0.199 | 5.10E-06 | 64 | 0.004 | 0.0005 | 6.74E-19 |  | | -0.0083 | 0.009 | 0.3276 |
| 20 | rs209961 | C | T | 0.722 | 6.42E-06 | 100 | 0.004 | 0.0004 | 1.38E-17 |  | | 0.0071 | 0.008 | 0.347 |
| 20 | rs3091842 | A | G | 0.044 | 6.81E-06 | 81 | -0.009 | 0.001 | 1.49E-18 |  | | -0.022 | 0.016 | 0.1648 |
| 20 | rs35666081 | A | G | 0.4 | 1.92E-06 | 25 | 0.002 | 0.0004 | 4.52E-08 |  | | 0.0023 | 0.007 | 0.732799 |
| 20 | rs6136492 | T | C | 0.226 | 3.15E-06 | 36 | -0.003 | 0.0005 | 5.88E-09 |  | | -0.0129 | 0.008 | 0.1029 |
| 22 | rs4820324 | C | G | 0.581 | 4.38E-06 | 56.25 | 0.003 | 0.0004 | 3.78E-14 |  | | -0.0133 | 0.007 | 0.0507598 |

AF, atrial fibrillation; SNP, single-nucleotide polymorphism; EA, effect allele; OA, other allele; EAF, effect allele frequency; se, standard error.

**Table 5 Details of studies and datasets used for MR analyses**

| **Exposure/Outcomes** | **Sample size** | **Cases/non-cases** | **Number of SNP** | **Ethnicity** | **Author** | **Consortium** | **Year** | **Units** | **ID** |
| --- | --- | --- | --- | --- | --- | --- | --- | --- | --- |
| AF | 1,030,836 | 60,620/970,216 | 33,519,037 | European | Nielsen JB | HUNT, deCODE, MGI, DiscovEHR, UK Biobank, and the AFGen Consortium | 2018 | NA | ebi-a-GCST006414 |
| Serum potassium | 64,979 | - | 9,851,867 | European | Ben Elsworth | UK Biobank | 2018 | SD | ukb-b-17881 |
| Serum magnesium | 64,979 | - | 9,851,867 | European | Ben Elsworth | UK Biobank | 2018 | SD | ukb-b-7372 |
| Serum calcium | 64,979 | - | 9,851,867 | European | Ben Elsworth | UK Biobank | 2018 | SD | ukb-b-8951 |
| Serum phosphate | 431,448 | - | 13,585,254 | European | Neale lab | UK Biobank | 2018 | mmol/L | ukb-d-30810_raw |

SNP, Single Nucleotide Polymorphism; AF, Atrial fibrillation; HUNT, The Nord-Trøndelag Health Study; MGI, the Michigan Genomics Initiative; NA, not available.


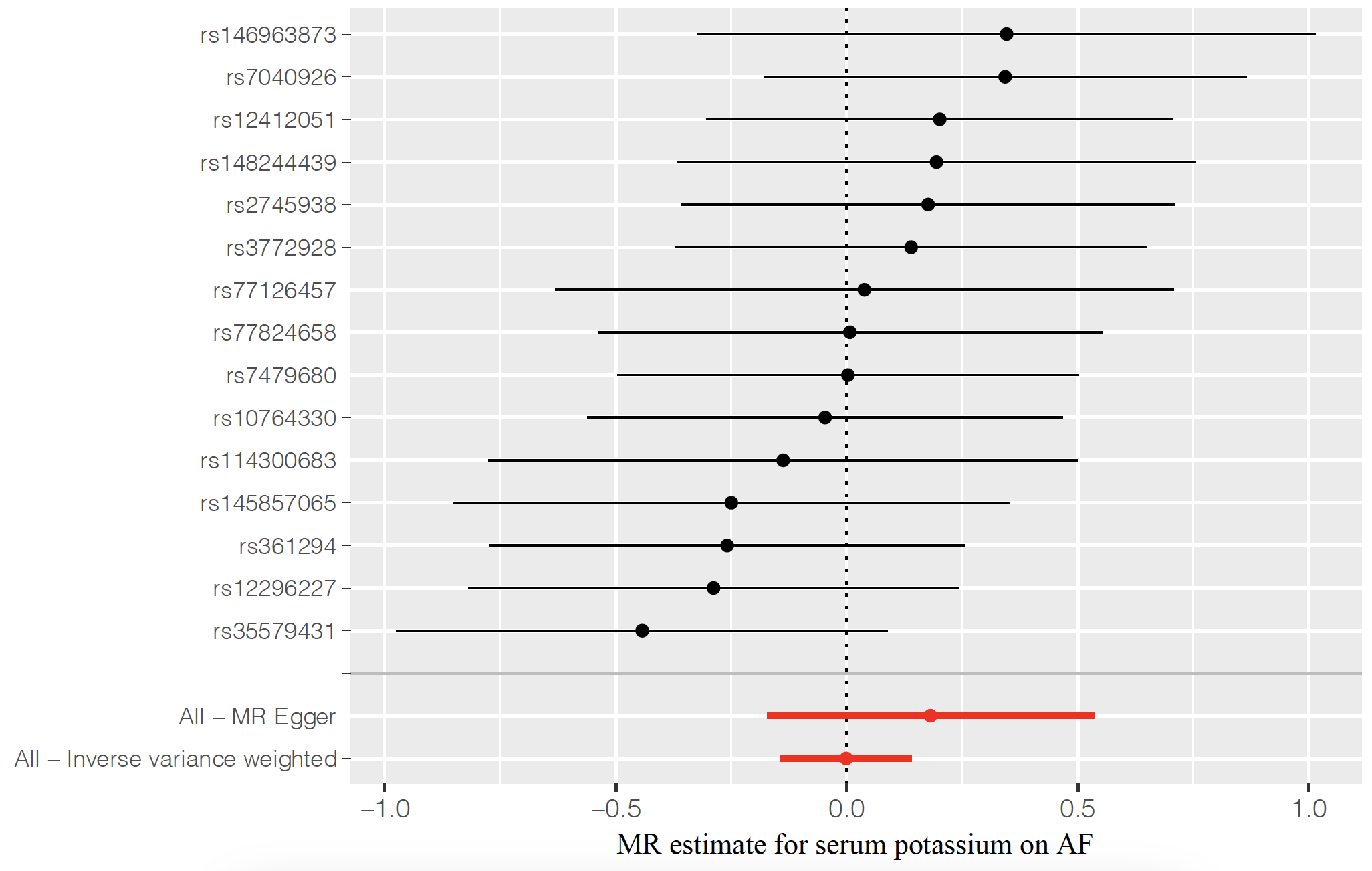


**Figure 1 Forest plot of causal effects between potassium-associated SNPs and risk of AF.**


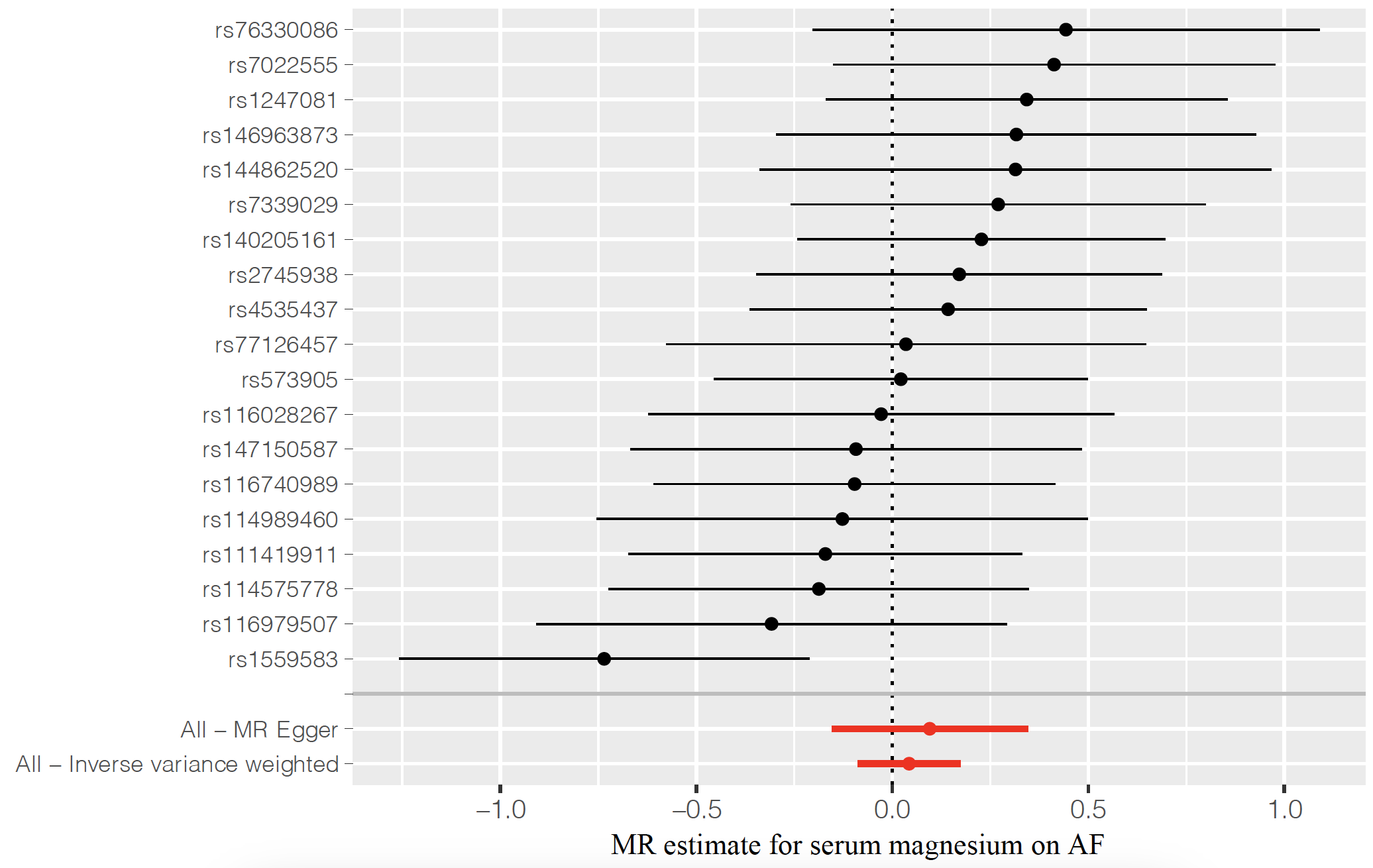


**Figure 2 Forest plot of causal effects between magnesium-associated SNPs and risk of AF.**


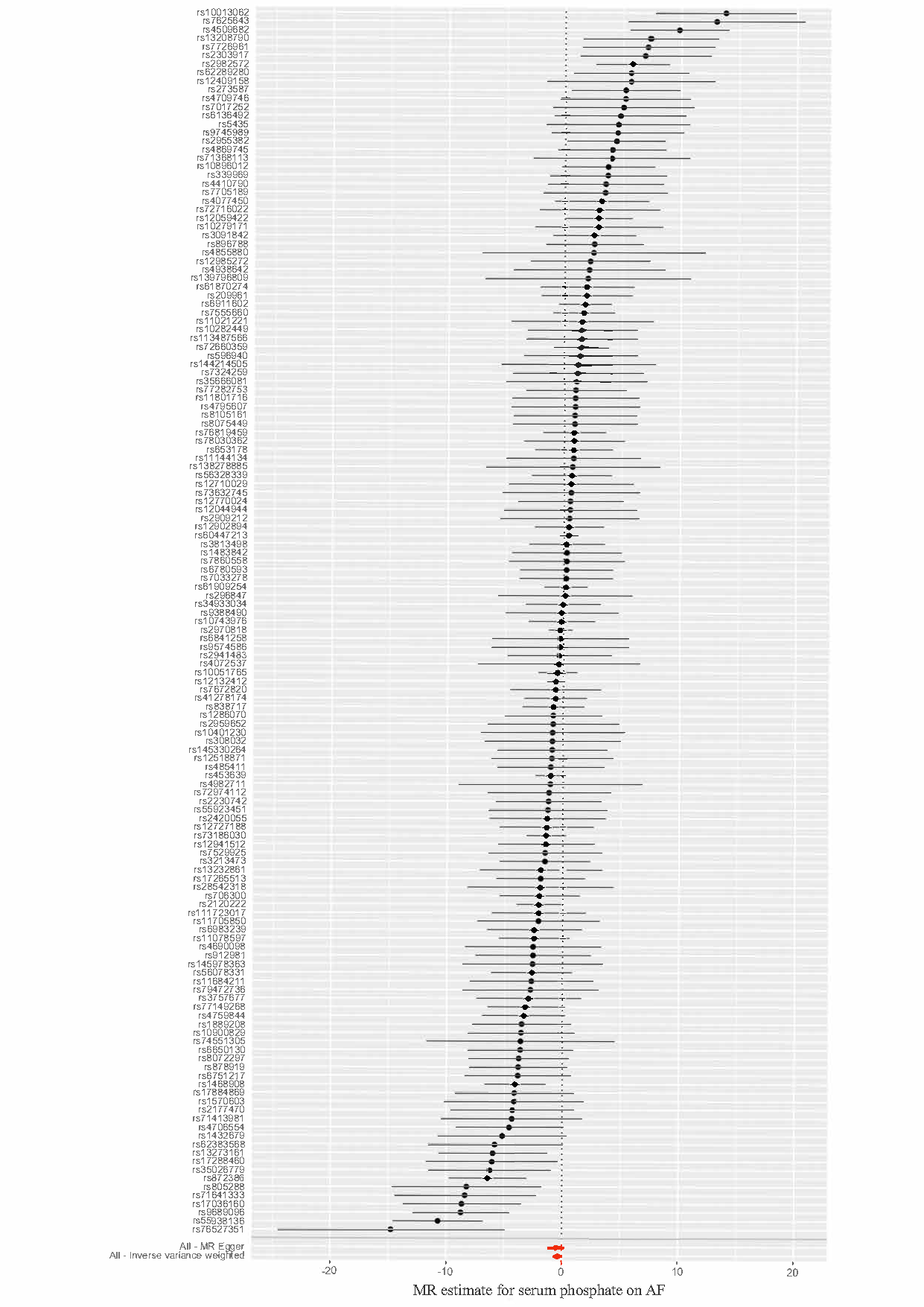


**Figure 3 Forest plot of causal effects between phosphate-associated SNPs and risk of AF.**


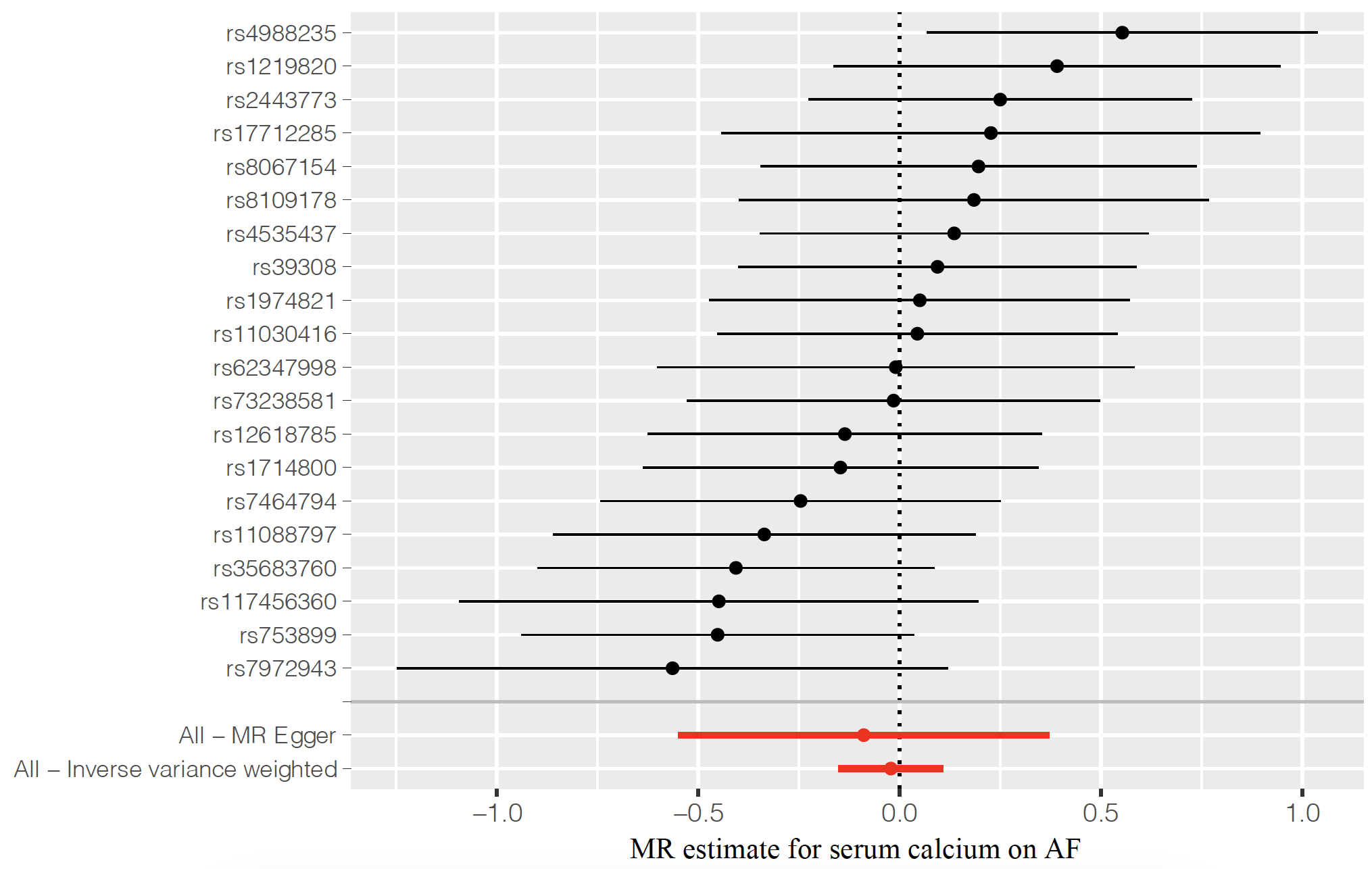


**Figure 4 Forest plot of causal effects between calcium-associated SNPs and risk of AF.**


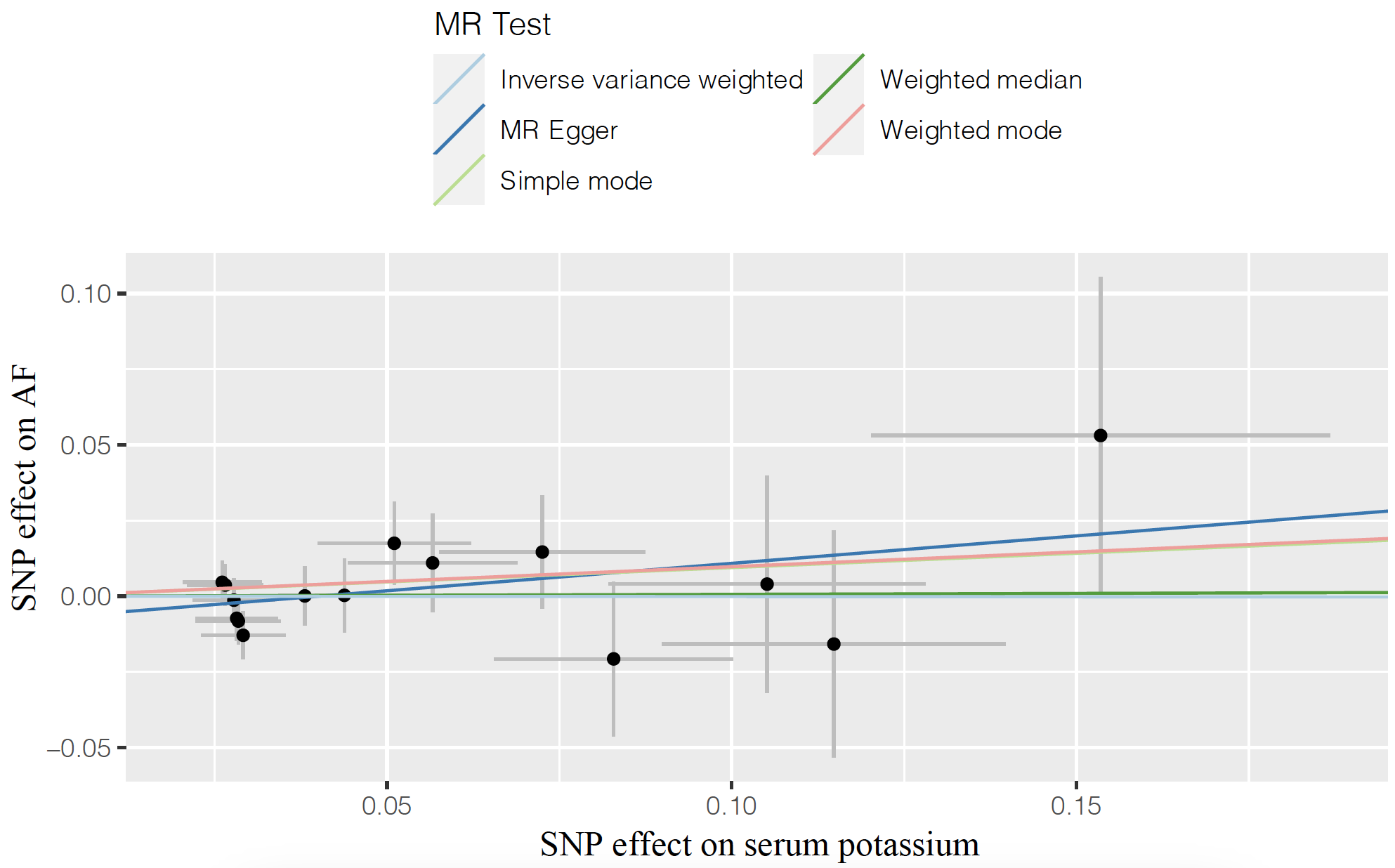


**Figure 5 Scatter plot of causal effects between potassium-associated SNPs and risk of AF.**


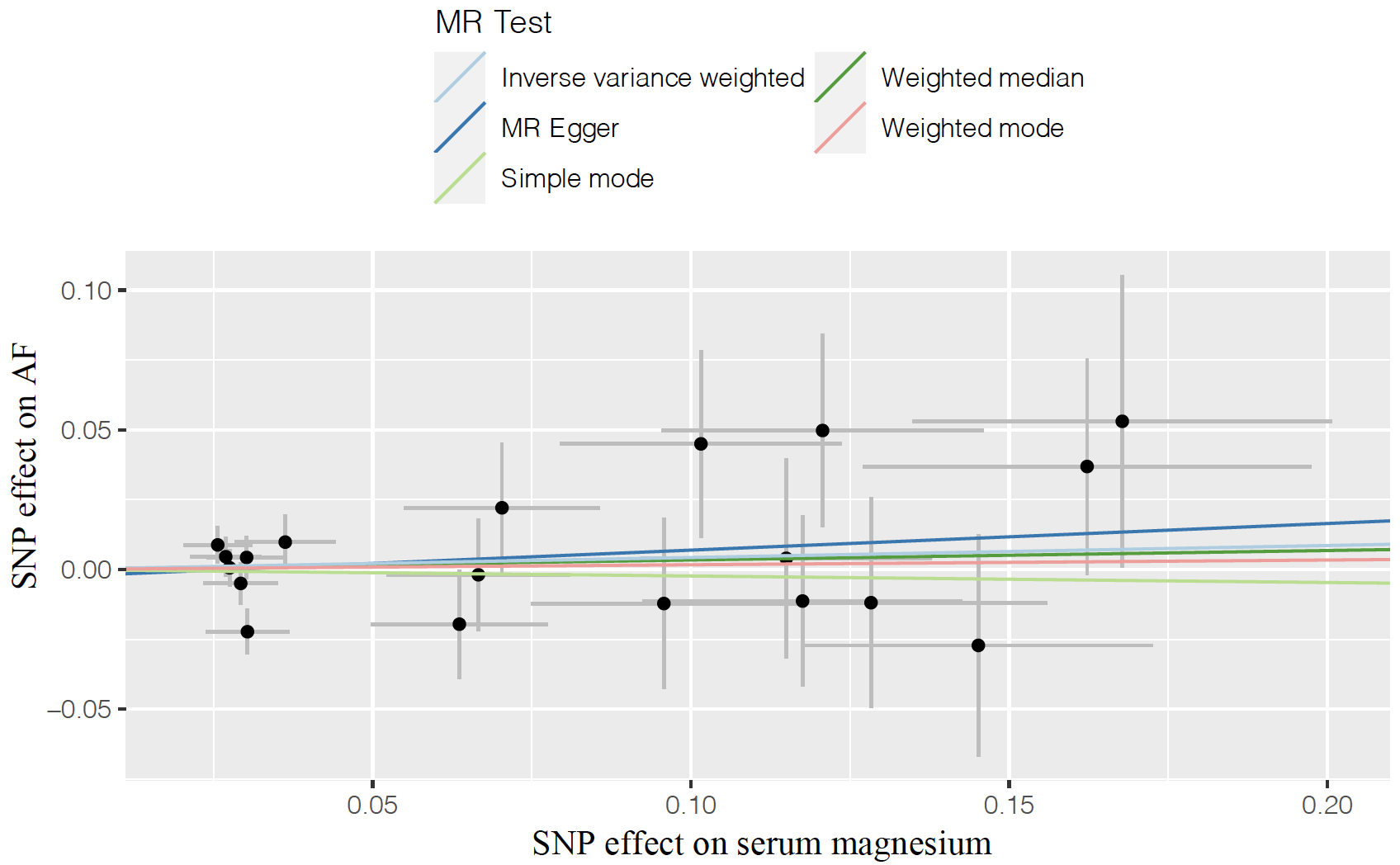


**Figure 6 Scatter plot of causal effects between magnesium-associated SNPs and risk of AF.**


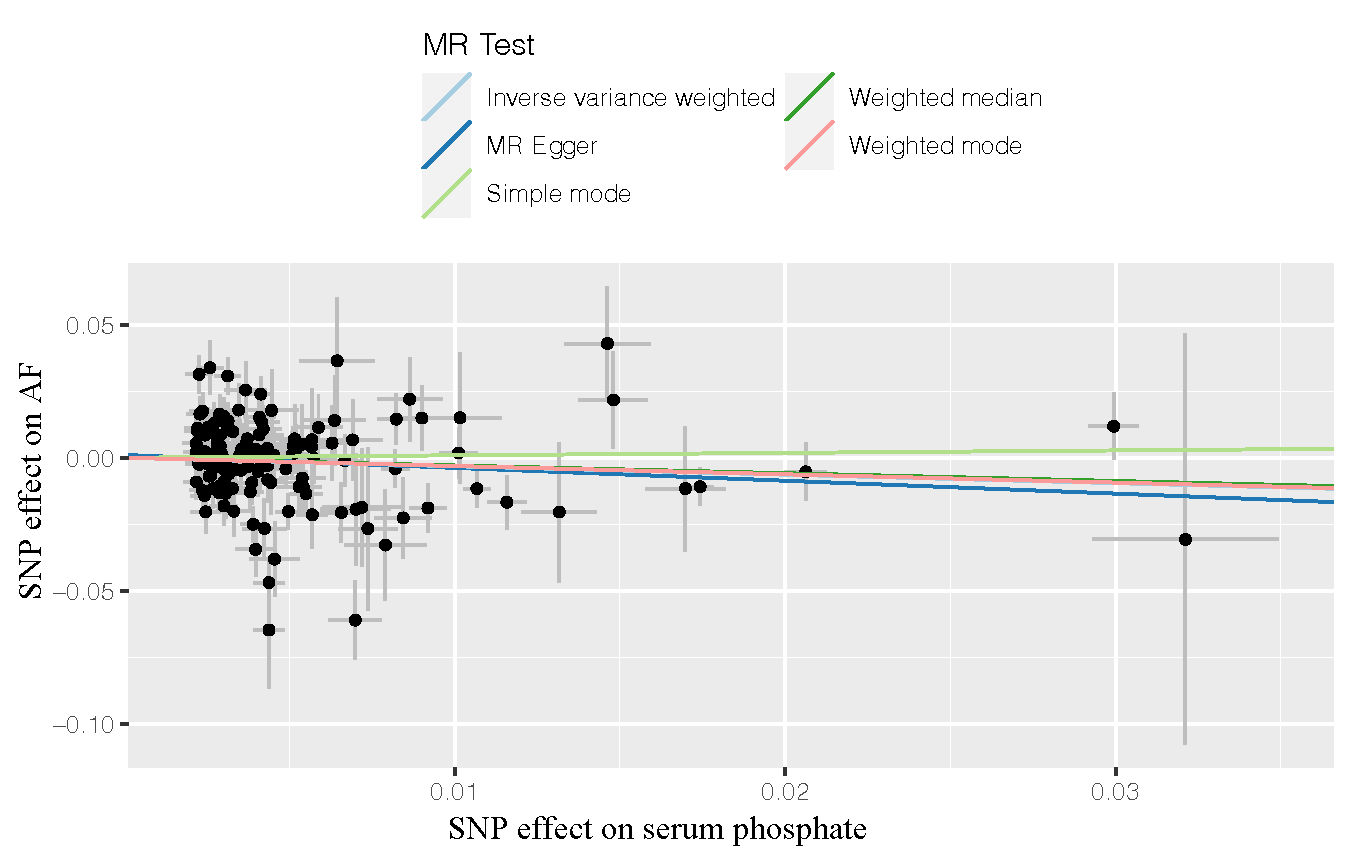


**Figure 7 Scatter plot of causal effects between phosphate-associated SNPs and risk of AF.**

**
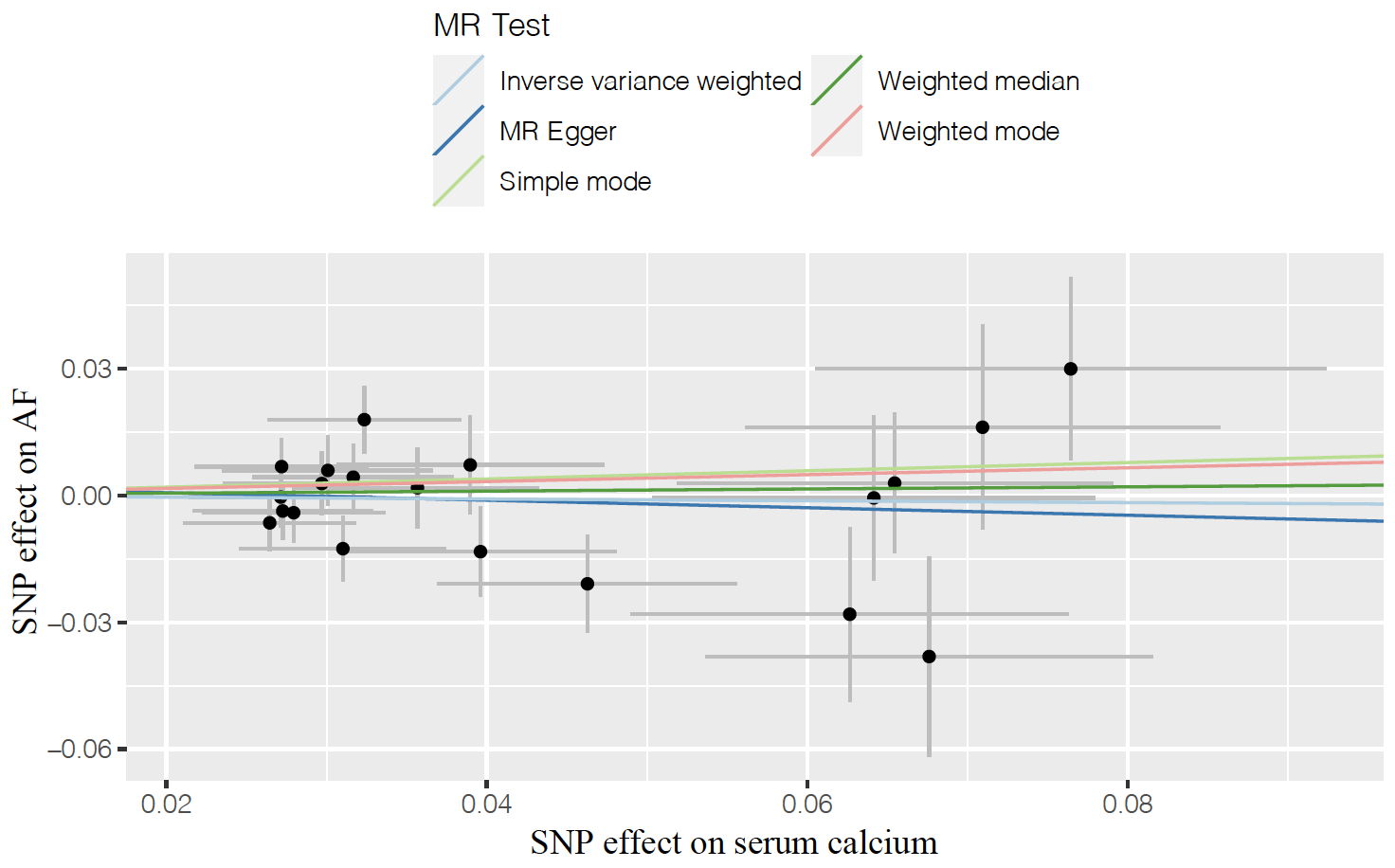
**

**Figure 8 Scatter plot of causal effects between calcium-associated SNPs and risk of AF.**


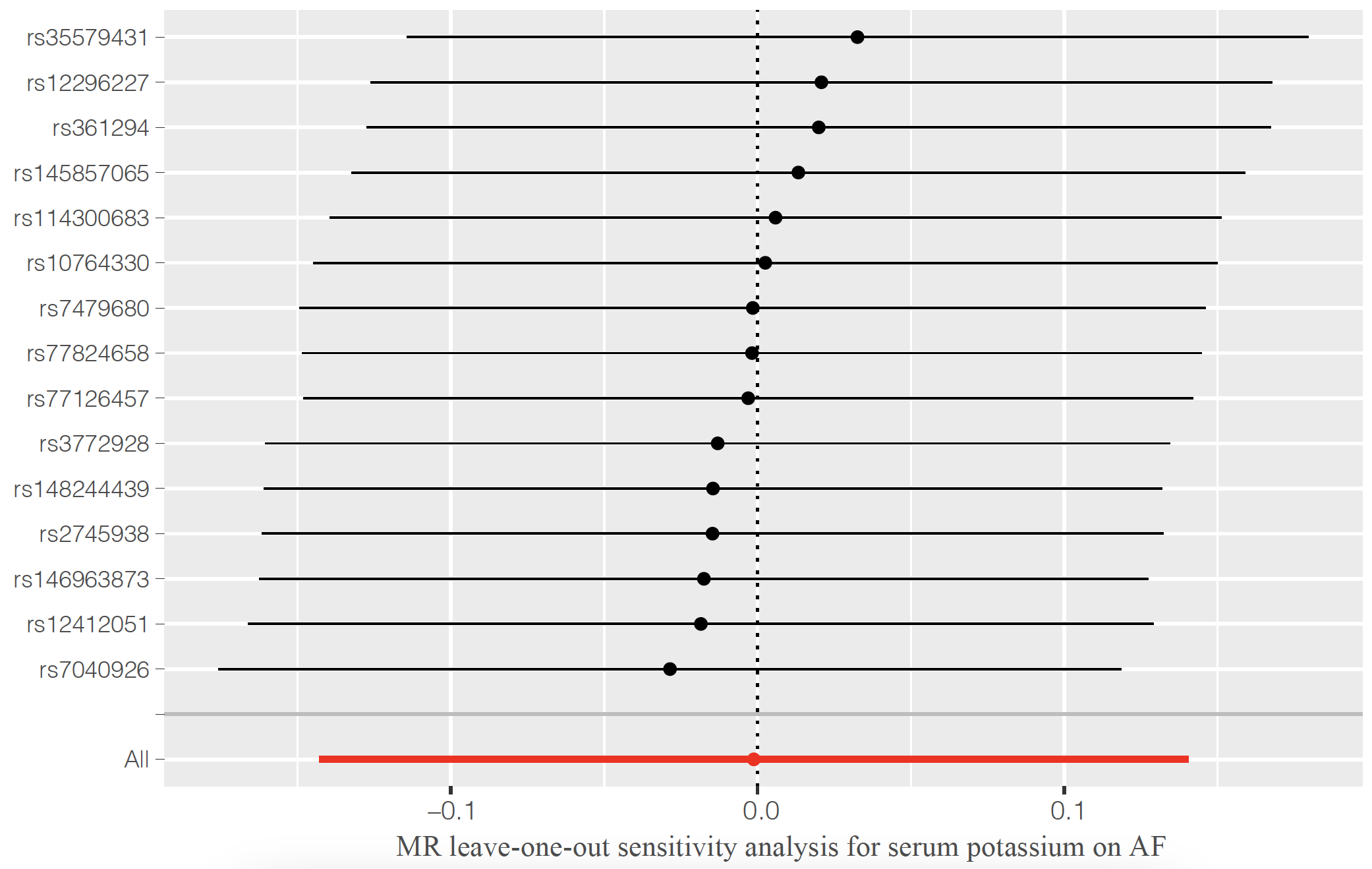


**Figure 9 MR leave-one-out sensitivity analysis for serum potassium on AF.**


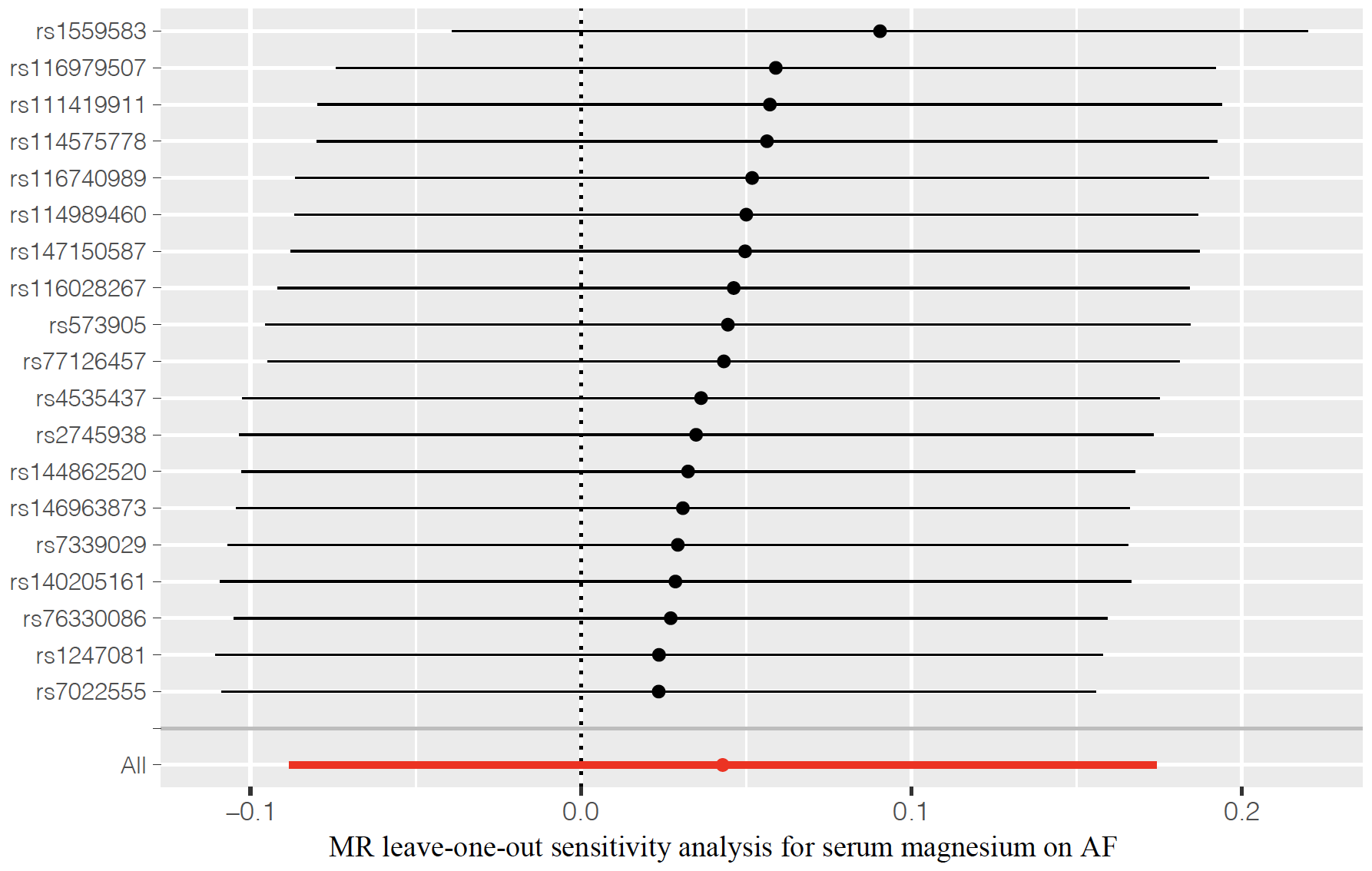


**Figure 10 MR leave-one-out sensitivity analysis for serum magnesium on AF.**


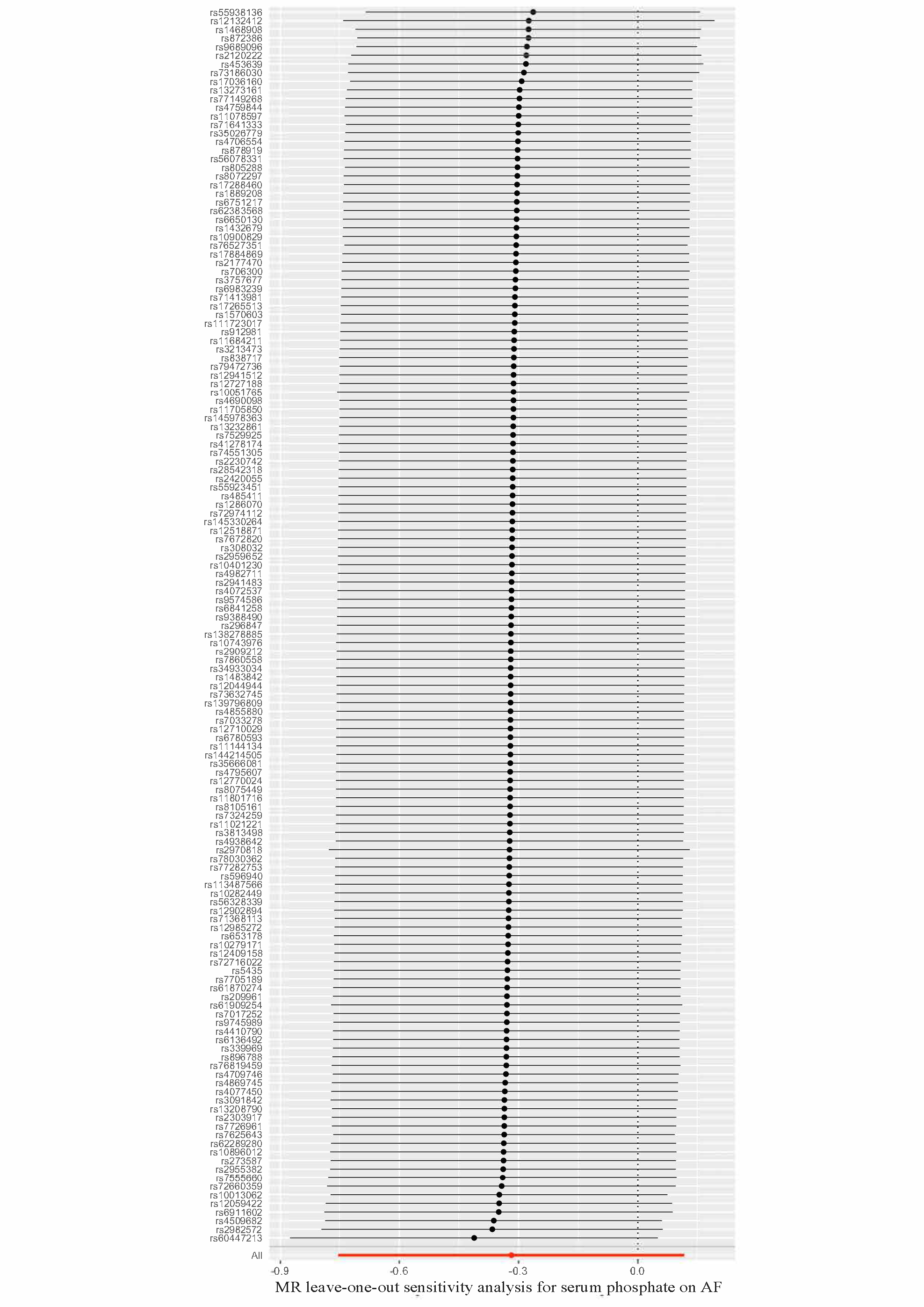


**Figure 11 MR leave-one-out sensitivity analysis for serum phosphate on AF.**


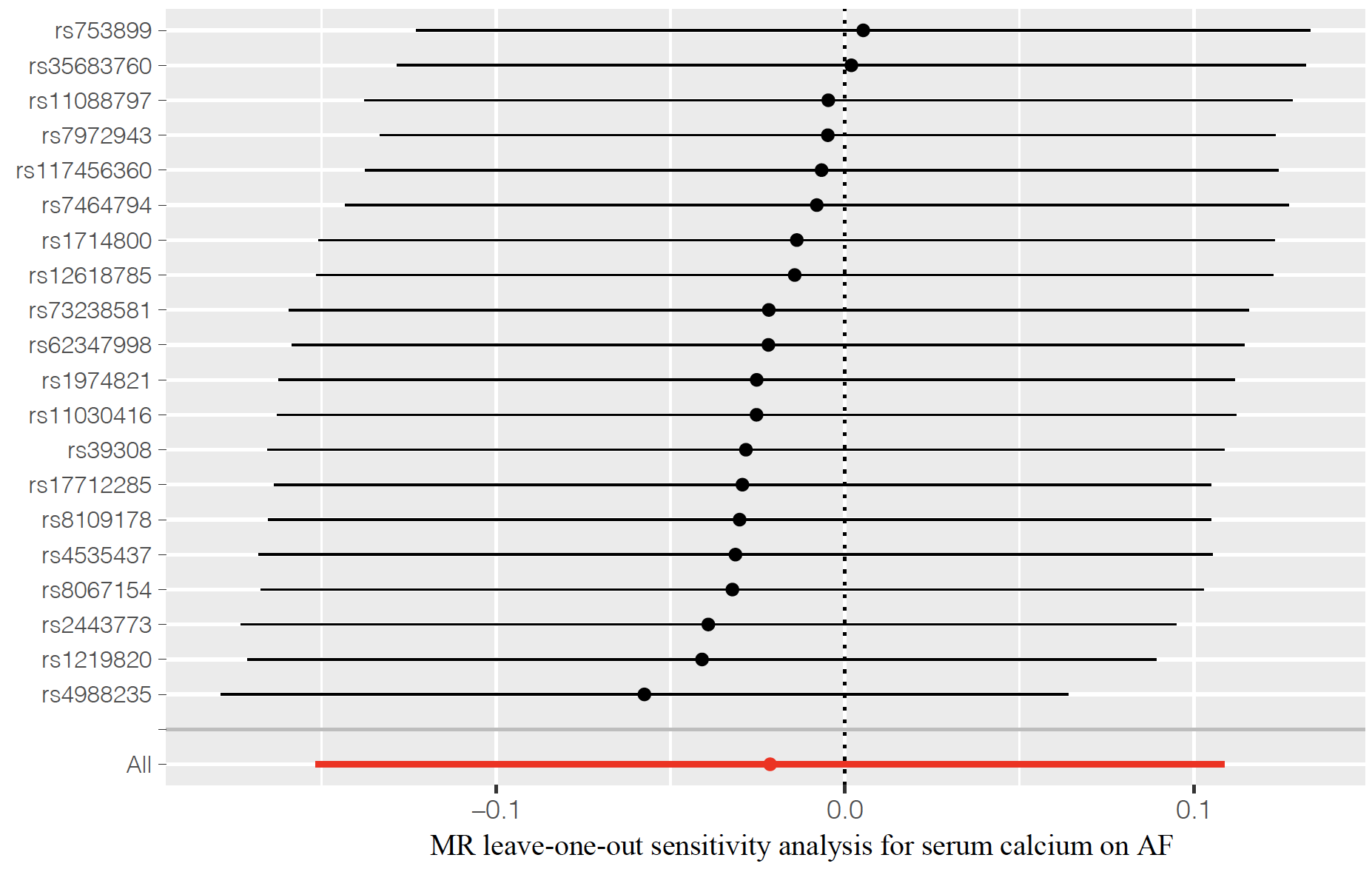


**Figure 12 MR leave-one-out sensitivity analysis for serum calcium on AF.**


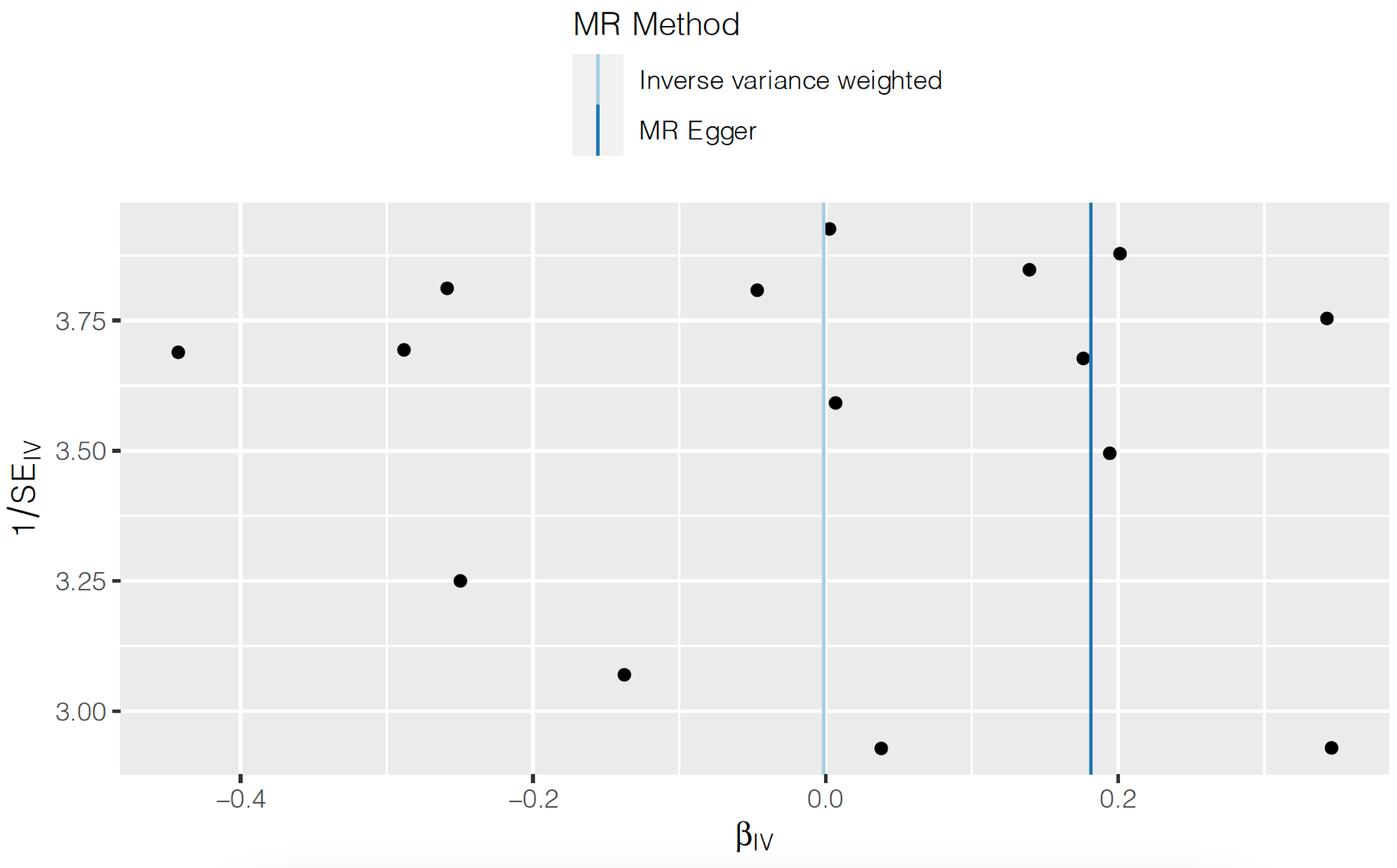


**Figure 13 Funnel plot of causal association between serum potassium on AF.**


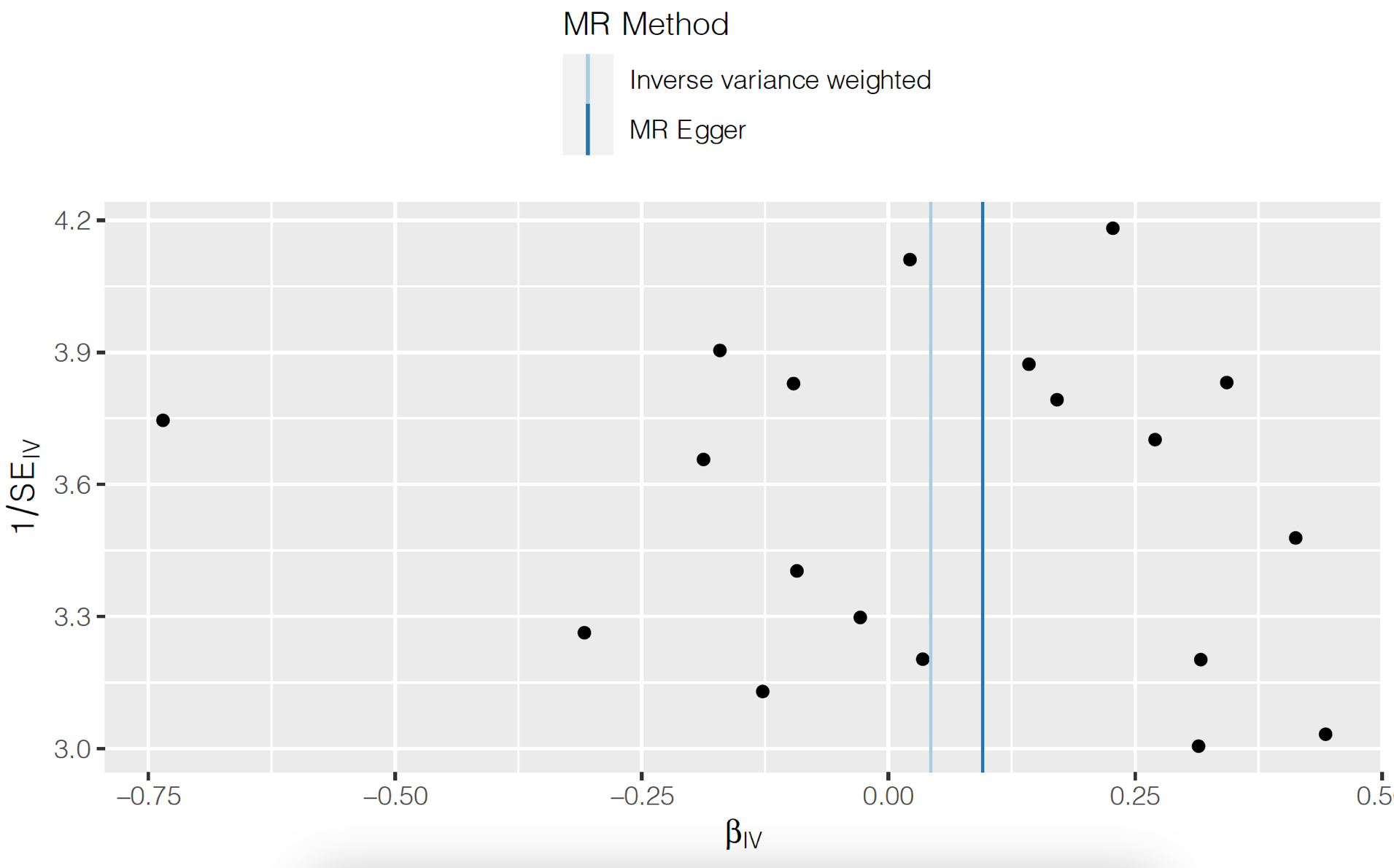


**Figure 14 Funnel plot of causal association between serum magnesium on AF.**


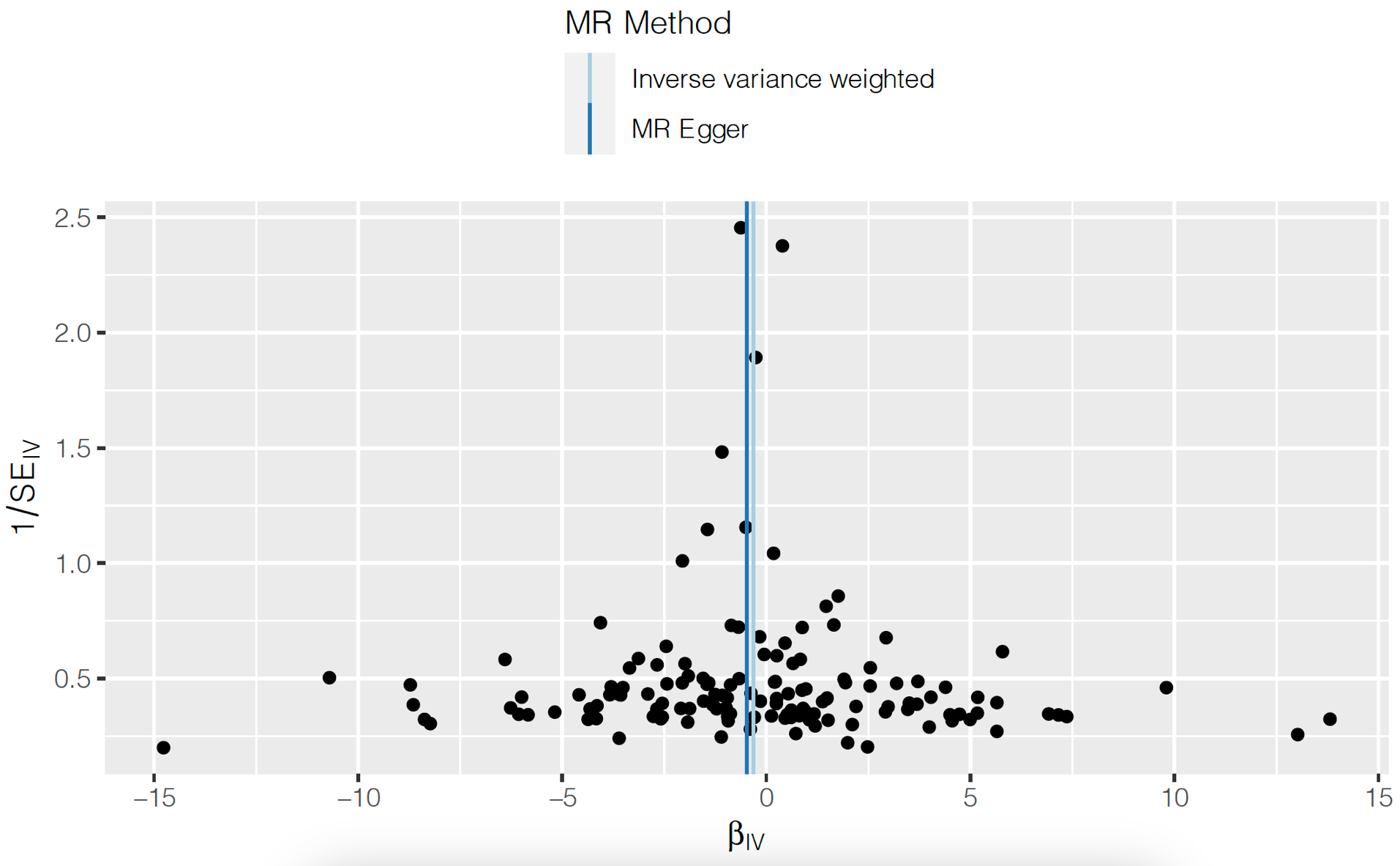


**Figure 15 Funnel plot of causal association between serum phosphate on AF.**


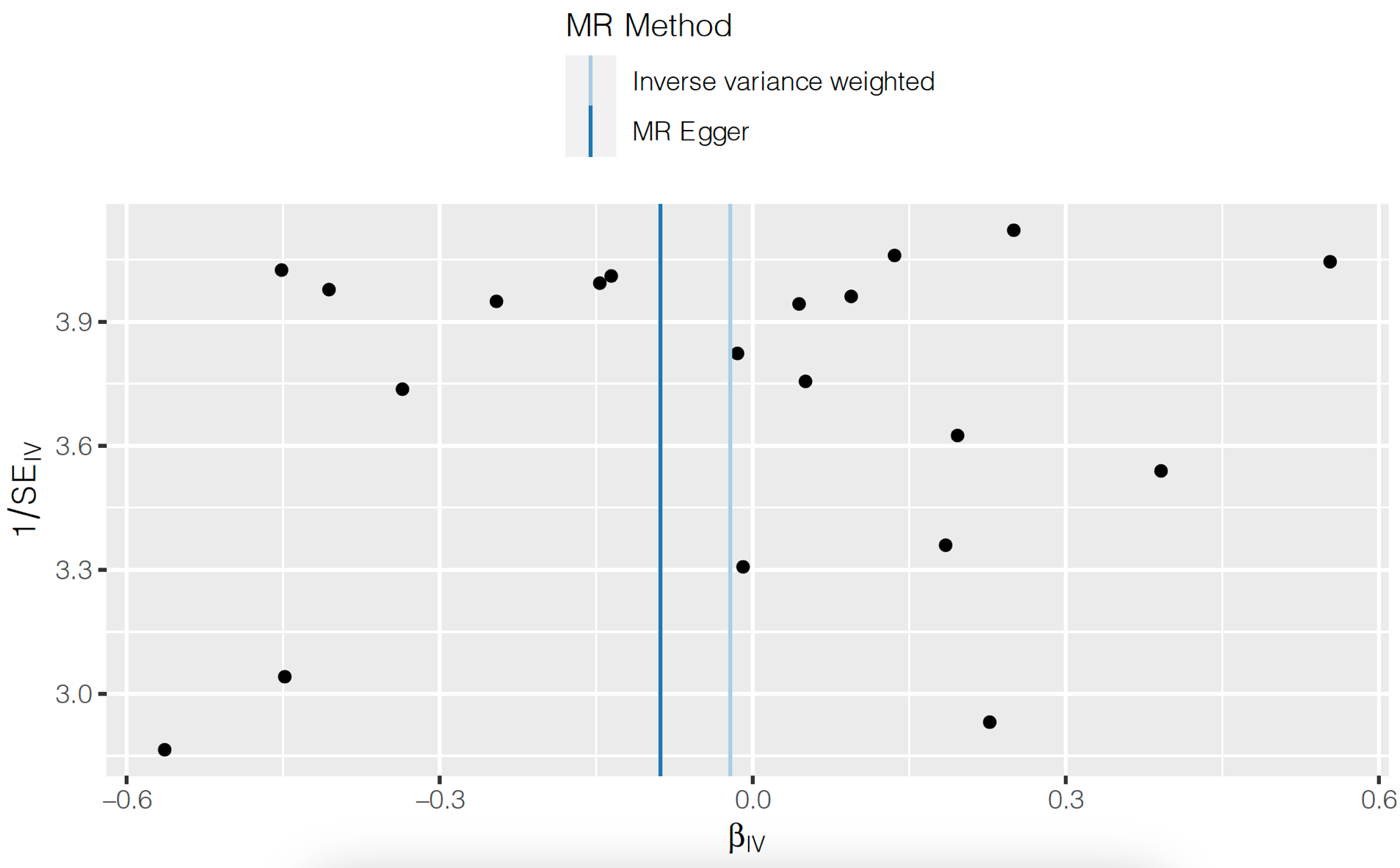


**Figure 16 Funnel plot of causal association between serum calcium on AF.**
